# Cleavage of Organosolv Lignin to Phenols Using Nitrogen Monoxide and Hydrazine

**DOI:** 10.1021/acsomega.1c00996

**Published:** 2021-07-23

**Authors:** Laura Elena Hofmann, Lisa-Marie Altmann, Oliver Fischer, Lea Prusko, Ganyuan Xiao, Nicholas J. Westwood, Markus R. Heinrich

**Affiliations:** † Department of Chemistry and Pharmacy, Pharmaceutical Chemistry, Friedrich-Alexander-Universität Erlangen-Nürnberg, Nikolaus-Fiebiger-Str. 10, 91058 Erlangen, Germany; ‡ School of Chemistry and Biomedical Sciences Research Complex, University of St. Andrews and EaStCHEM North Haugh, St Andrews KY16 9ST, Fife, United Kingdom

## Abstract

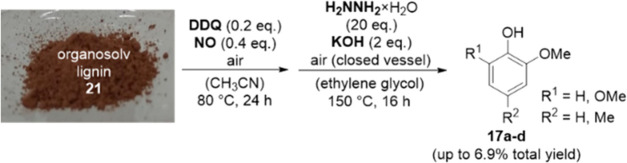

From the variety of methods known for the depolymerization of organosolv lignin, a broad range of diversely substituted aromatic compounds are available today. In the present work, a novel two-step reaction sequence is reported, which is focused on the formation of phenols. While the first step of the depolymerization strategy comprises the 2,3-dichloro-5,6-dicyano-1,4-benzoquinone (DDQ)-catalyzed oxidation of organosolv lignin with nitrogen monoxide so that two waste materials are combined, cleavage to the phenolic target compounds is achieved in the second step employing hydrazine and potassium hydroxide under Wolff–Kishner-type conditions. Besides the fact that the novel strategy proceeds via an untypical form of oxidized organosolv lignin, the two-step sequence is further able to provide phenols as cleavage products, which bear no substituent at the 4-position.

## Introduction

As a plant-derived biopolymer, lignin represents one of the major constituents of biomass besides cellulose and hemicellulose. Its main task is to provide sufficient rigidity to the cell walls of plants. In native lignin, which is largely colorless, the molecular weight of the oligomers and polymers may range from 1000 to 20 000 g/mol.^1^ After acidic or alkaline treatment, which may be applied prior to a depolymerization process, the color of lignin typically changes to dark brown.^2^ In 2014, the overall lignin market in the world amounted to around U.S. $775 million and estimations foresee an increase to U.S. $900 million by the year 2020.^1^ As the major side product in the pulp and paper industry,^3^ lignin represents one of the most readily available natural polymers,^4^ although only around 2% of the 50 million tons of lignin were used for the production of chemicals in 2010. The remaining amount, and thus the vast majority, is transferred to power and heat generation.^5^

**Figure 1:**
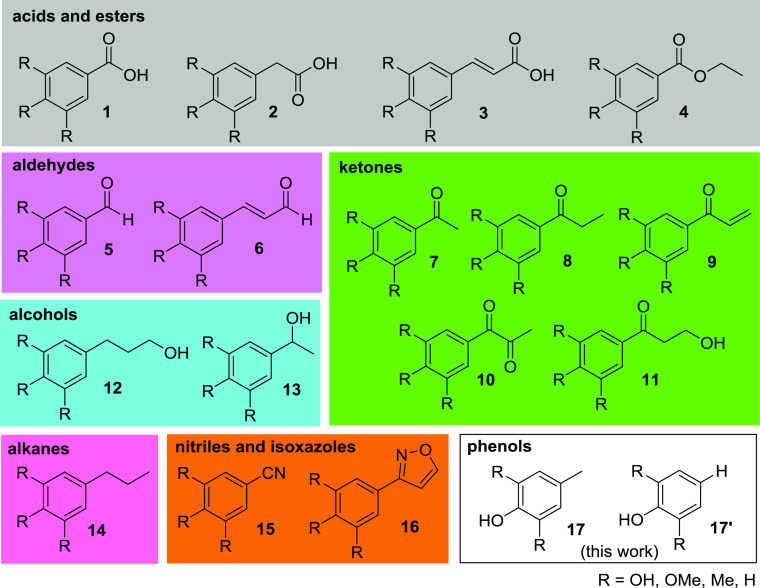
Overview of major products **1**–**17** obtained from one- and two-step cleavage processes of lignin.

Against this background, the valorization of lignin, although being a challenging task due to its complex molecular structure, is an important objective in the field of sustainable chemistry,^6^ and many strategies are known today to promote lignin depolymerization. Such strategies include oxidative treatment,^7^ solid-acid-catalyzed methods,^8^ as well as high-temperature-based methods.^9^ Furthermore, two-step procedures have become increasingly prominent, such as transition-metal-catalyzed^10^ or biocatalytic methods.^11^ The most intensively investigated two-step lignin depolymerization procedures typically consist of a first oxidation step, which in most cases oxidizes the benzylic alcohol in the β–O–4 linkage to an aryl ketone, followed by a second, reductive cleavage step of the adjacent ether bridge. Such procedures usually lead to compounds of the general structures **1**–**11** (Figure 1), when oxidants such as 2,3-dichloro-5,6-dicyano-1,4-benzoquinone (DDQ)/*t*BuONO, DDQ/HNO_3_, or (2,2,6,6-tetramethylpiperidin-1-yl)oxyl (TEMPO)/HNO_3_ and reductants such as formic acid or zinc are employed.^12^ Depending on the selection of the reductant (e.g., TiN–Cu nanoparticles, Bi_2_MoO_6_/CdS composites), the formation of additional or exclusive compounds of the general structure **13**(13) or **15** and **16**(14) is observed. Compounds of structures **12** and **14**, in contrast, are rarely obtained by two-step oxidation–reduction procedures.^15^ Procedures which produce 4-methyl substituted phenols **17** and especially 4-unsubstituted phenols **17′** in significant amounts do exist; however, they follow different lignin valorization strategies such as alkaline depolymerization of Kraft lignin^16^ or gas-phase hydroprocessing of lignin oil.^17^

Our approach to the cleavage of lignin is based on employing nitrogen monoxide^18,19^ in the oxidation step, since this would allow us to combine the use of two waste materials, lignin and nitrogen monoxide, in a unique valorization process. In particular due to their acute toxicity, nitrogen monoxide^18a^ and dioxide^20^ range among the most dangerous waste gases resulting from large-scale combustion as well as several other industrial processes.^21^ The variety of these processes, which include nitric acid manufacturing, preparation of fertilizers and explosives, as well as metallurgical processes, glass manufacturing and cement kilns, however, also shows that nitrogen oxides are quite well controllable. The required removal of the waste gases, which is commonly termed denitrification, is currently achieved through two main strategies known as selective catalytic reduction (SCR)^22^ and selective noncatalytic reduction (SNCR).^23^ If applicable for the depolymerization of lignin, nitrogen monoxide would thus not only serve as a cheap reagent but also as an oxidant, which would be favorable to be used as an alternative to the above-mentioned established methods for denitrification.

**Scheme 1:**
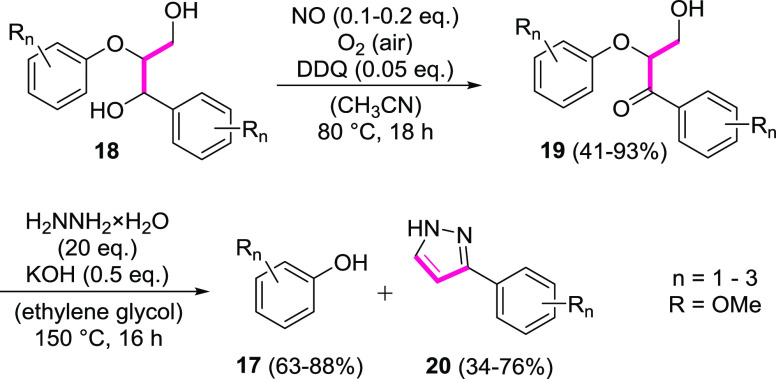
Two-Step Strategy for the Cleavage of Lignin Systems Using Nitrogen Monoxide in the Oxidation and Hydrazine in the Reduction Step

After a first successful application of our nitrogen monoxide-based two-step strategy to established lignin systems **18** (Scheme 1),^24^ where the redox-neutral cleavage of the oxidized ketone intermediate **19** was performed with hydrazine under Wolff–Kishner conditions, we now turned to an application of this protocol to organosolv lignin and further lignin variants.

In the present work, we show that the two-step combination of nitrogen monoxide and hydrazine, when applied to organosolv lignin, does not lead to phenols **17** and pyrazoles **20**, as observed before for the lignin systems **18**, but only to phenols **17** as major cleavage products. Combined with the fact that the reactions proceed via an untypical form of oxidized organosolv lignin, and a yet unknown C–C bond cleavage is required for product formation, these results shed light on promising but so far unexploited opportunities in lignin depolymerization.

## Results and Discussion

In the previous study on the cleavage of lignin systems (Scheme 1),^24^ only low amounts of nitrogen monoxide (0.1–0.2 equiv) in combination with catalytic 2,3-dichloro-5,6-dicyano-*para*-benzoquin-one (DDQ) (0.05 equiv) had been sufficient for the oxidation step. While the degree of oxidation of the lignin systems could be readily monitored by ^1^H NMR spectroscopy, the oxidation of the biopolymer is more difficult to follow and two-dimensional (2D) NMR techniques such as heteronuclear single quantum coherence (HSQC) NMR spectra are typically applied.^11,12d,15,25^

**Figure 2:**
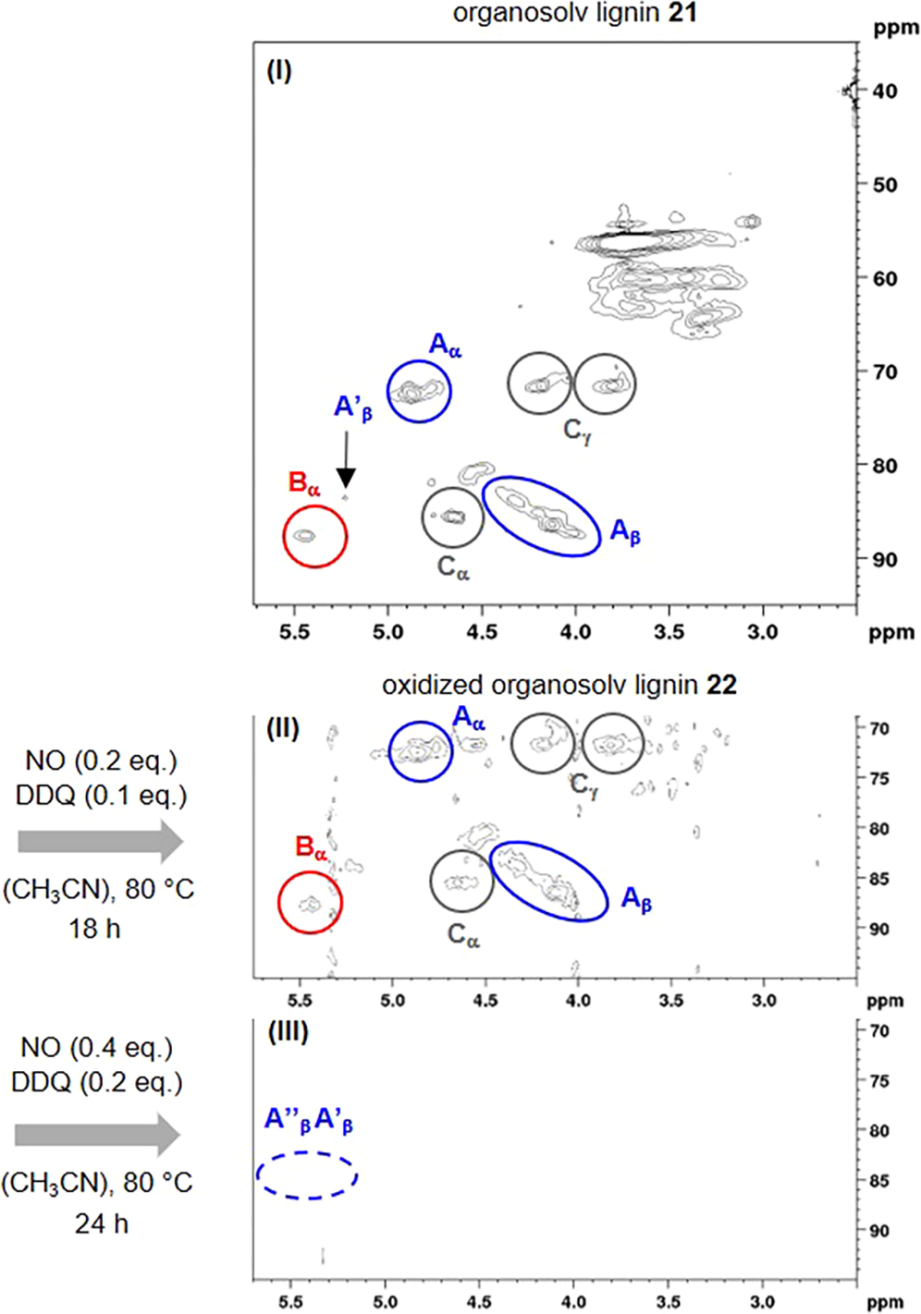
Partial 2D HSQC NMR spectra before (I) and after (II, III) oxidation of organosolv birch lignin **21** by nitrogen monoxide in the presence of DDQ under air.

To investigate the applicability of our reaction conditions comprising nitrogen monoxide and catalytic DDQ, a short series of optimization experiments was carried out (Figure 2), whereby the amounts (and equiv) of nitrogen monoxide and DDQ were calculated based on a rounded average mass of 200 g/mol for one lignin monomer. This average value was obtained from the weighted average of the molecular weights of coniferyl (180 g/mol) and sinapyl (210 g/mol) alcohols present as lignin monomers, where an analysis of the starting material led to a ratio of sinapyl to coniferyl monomers of 2.73:1. This ratio corresponds to a precise average mass of 202 g/mol. Further analysis of the starting material showed that the content of β–O–4 linkages is around 24% (see the Supporting Information).

**Figure 3:**
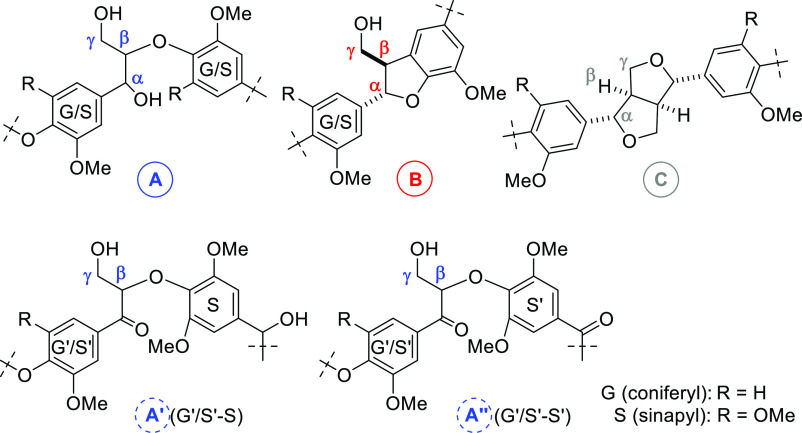
Representative nonoxidized (**A**–**C**) and oxidized (**A′**, **A″**) fragments of the organosolv lignin structure.^9c,12d^

Upon variation of the amounts of nitrogen monoxide, DDQ, and the reaction time, the 2D HSQC NMR spectra depicted in Figure 2 were obtained. All oxidation reactions were carried out under otherwise identical conditions, using 1 g of organosolv birch lignin **21** in acetonitrile at a reaction temperature of 80 °C in a closed vessel under air. For assignment of the cross-peaks appearing in the HSQC NMR spectra, characteristic fragments from organosolv lignin are shown in Figure 3, where the abbreviations G and S refer to coniferyl- and sinapyl-alcohol-derived units, respectively.

Particularly useful NMR signals to investigate the degree of oxidation are the protons in the α, β, and γ positions of the fragments **A**–**C** (Figure 3), which are typically shifted or disappear upon oxidation. The relevant cross-peaks in the HSQC NMR spectrum are mainly located δ = 3.5–5.5 ppm in the ^1^H dimension and δ = 70–90 ppm in the ^13^C dimension (Figure 2).

While an attempt with 0.2 equiv of nitrogen monoxide and 0.1 equiv of DDQ left a considerable amount of the native β–O–4 units intact after 18 h (cf. spectra **I** and **II**, Figure 2), a comparably strong oxidation of organosolv lignin **21** was observed using 0.4 equiv of nitrogen monoxide and 0.2 equiv of DDQ (cf. spectra **I** and **III**, Figure 2). At a prolonged reaction time of 24 h, these conditions led to the complete disappearance of the cross-peaks assigned to the benzylic α positions (A_α_, B_α_, C_α_) and also of the signals related with the more distant A_β_ and C_γ_ positions. However, characteristic signals previously assigned to the β positions of the oxidized lignin fragments **A′** and **A″**(12d) could not be detected in spectrum **III**. These cross-peaks for the A_β_′ and A_β_″ positions should appear at δ = 5.2–5.7 ppm (^1^H) and δ = 85 ppm (^13^C). As a consequence, the reaction conditions including nitrogen monoxide and DDQ apparently lead to an oxidized form **22** of organosolv lignin, which is different from that resulting from an oxidation by *tert*-butyl nitrite in the presence of DDQ, as the latter method has been confirmed to provide oxidized fragments such as **A′** and **A″**.^12d^ Notably, these results also differ from the previous oxidation of the lignin systems **18** (Scheme 1), where a comparably selective oxidation of the benzylic alcohol unit to the corresponding ketone was observed. The full spectra, from which the sections **I**–**III** depicted in Figure 2 were cut, are shown in the Supporting Information. These confirm that the characteristic cross-peaks labeled in sections **I** and **II** (Figure 2) do not move to another area of the spectrum but disappear, which in turn indicates a strong oxidative impact on the aliphatic subunits.

**Scheme 2:**
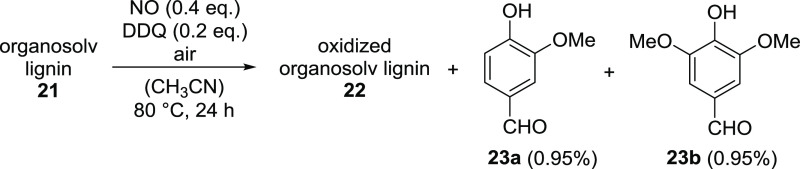
Oxidation of Organosolv Lignin **21** with Nitrogen Monoxide in the Presence of DDQ under Air

Due to the complete absence of signals in the characteristic region of the HSQC NMR spectrum (see spectrum **III**, Figure 2), it was unclear at this point whether the untypical oxidation by nitrogen monoxide and DDQ had already led to a partial depolymerization of organosolv lignin. To investigate this aspect, the oxidized organosolv lignin **22** was submitted to column chromatography. While the significant difficulties encountered with solubilization point to the fact that the oxidized lignin **22** has remained largely polymeric, two aldehydes, vanillin (**23a**) and syringaldehyde (**23b**), could be separated and identified.^7a,7b,9a,9b,12b,26^ The oxidation step can thus be described as shown in Scheme 2, where the yields of **23a** and **23b** were calculated on the basis of the estimated average mass of 200 g/mol per lignin monomer (see above). As an example, the yields from an oxidation experiment with 1 g of organosolv lignin **21** refer to 5 mmol of starting material.

**Scheme 3:**
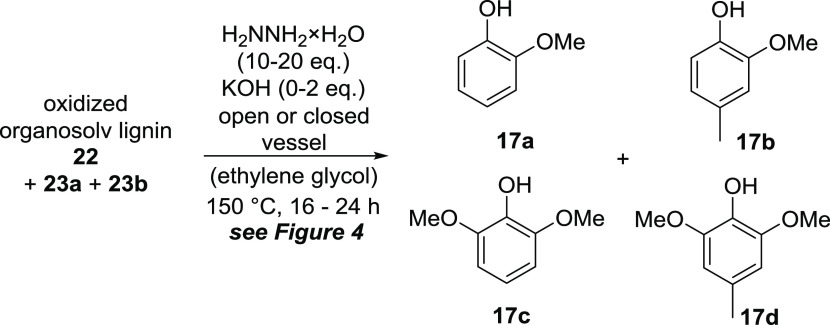
Variation of Conditions during Optimization of the Hydrazine-Mediated Cleavage of Oxidized Organosolv Lignin **22**

In the next step, we turned to evaluate the cleavage of the oxidized organosolv lignin **22** using hydrazine as a reductant under Wolff–Kishner conditions. The first attempt was carried out under the cleavage conditions previously optimized for the lignin systems (Scheme 1) applying hydrazine hydrate (20 equiv) and potassium hydroxide (0.5 equiv) in ethylene glycol at 150 °C for a reaction time of 24 h. An analysis of the product mixture revealed the formation of four phenols **17a**–**d** (Scheme 3) in a total yield of 4.1% (**17a**: 1.1% **17b**: 1.2% **17c**: 1.4% **17d**: 0.4%) based on the initially used amount of nonoxidized organosolv lignin **21** (yields over two steps). Further investigation of the remaining polymeric material by NMR however showed no defined or assignable substructures.

Aryl pyrazoles **20**, which had been identified as cleavage products from lignin systems **18** in our previous study (Scheme 1), were not detected in the crude product mixture resulting from the treatment of oxidized lignin **22** with hydrazine (Scheme 3). Together with the untypical oxidation to **22** discussed above, this shows that lignin systems such as **18**, although well established, are not necessarily capable of predicting the outcome with structurally more complex, natural polymers.

**Figure 4:**
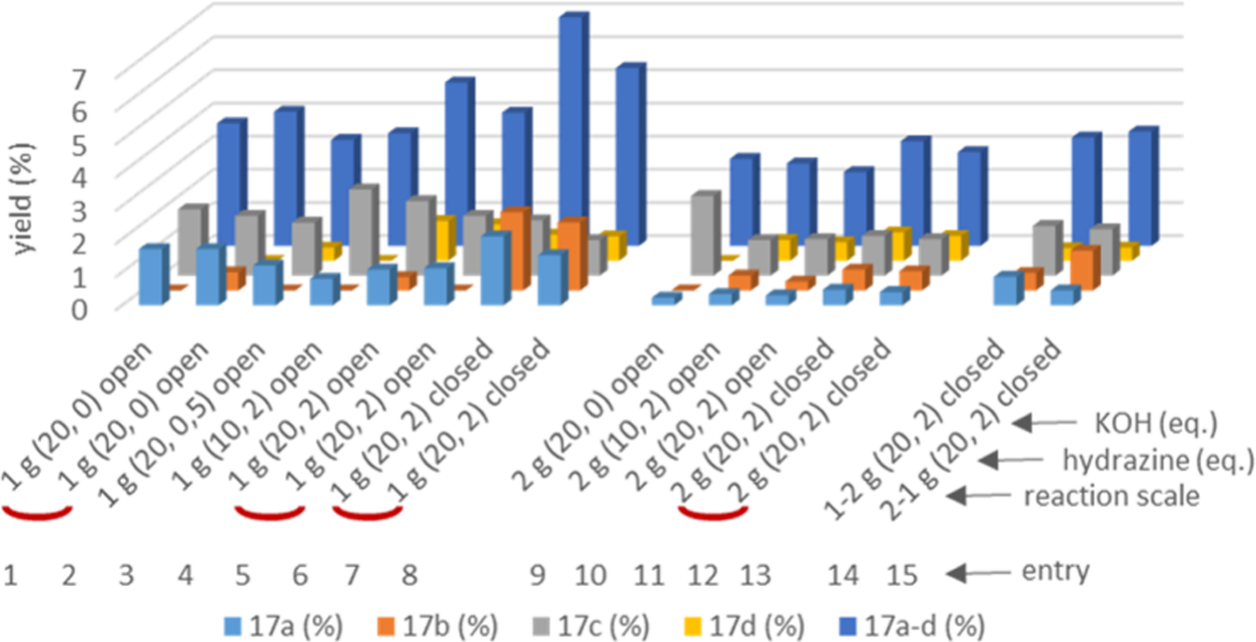
Hydrazine-mediated cleavage of oxidized organosolv lignin **22** further containing aldehydes **23a** and **23b**. Yields determined after purification by column chromatography using maleic acid as internal standard. Yields based on organosolv lignin **21** (yields over two steps).

The results from selected experiments aimed at the optimization of the cleavage step are summarized in Figure 4. The variation of parameters was thereby focused on the amounts of hydrazine and potassium hydroxide as well as the effect of access to open air. Reactions designated as “open” were carried out with an open reflux condenser, reactions labeled as “closed” were also performed under air, but only equipped with a balloon for pressure equilibration. Otherwise, all experiments shown in Figure 4 were run at 150 °C with a reaction time of 16 h (see the Supporting Information). Note that all entries in Figure 4 result from a two-step sequence, meaning that the oxidation step was carried out individually before the hydrazine-mediated cleavage. This approach was chosen to evaluate the reproducibility of the whole sequence.

In the series of optimization, where the 1 and 2 g scales refer to the initial amount of organosolv lignin **21** used for the two-step sequence, the “closed” reaction conditions turned out as the more favorable for the reduction step (Figure 4). Within the 1 g series, the change from “open” to “closed” at 20 equiv of hydrazine and 2 equiv of potassium hydroxide (entries 5–8) led to a strong increase of phenol **17b**, which can also be identified, but not as significant, on the 2 g scale (entries 11–13). The most selective reaction was observed on the 2 g scale with 20 equiv of hydrazine and in the absence of potassium hydroxide (entry 9), as this reaction gave almost only phenol **17c** (2.4%), while under similar conditions on the 1 g scale, a mixture of **17a** (1.7%) and **17c** (1.8–2.0%) was obtained (entries 1 and 2).

In general, reactions conducted under identical conditions (four pairs, entries 1, 2, 5–8, 12, 13) turned out as reasonably reproducible, but they still show deviations. More surprising are, however, the strong deviations when changing from the 1 g scale to the larger 2 g scale. Besides the points discussed above, and the fact the yields were roughly halved when doubling the scale (cf. entries 7 and 8 with 12 and 13), also the product distribution pattern under the so far optimal conditions was almost reversed. While at the 1 g scale “closed”, the highest yield was obtained for **17b** (followed by **17a**, **17c**, and **17d**; see entries 7 and 8), the identical conditions on the 2 g scale provided **17c** as the most abundant phenol followed by **17d**, **17b**, and **17a** (entries 12 and 13).

To get further insights into which one of the two steps in the sequence would be responsible for the decrease in yield, two mixed experiments were conducted. On the one hand, oxidation on the 1 g scale (performed twice) was combined with reduction on the 2 g scale (entry 14), and vice versa (entry 15). The slight increase in overall yield observed for these two mixed experiments (entries 14 and 15), compared with the best 2 g experiments (entries 12 and 13), supports the assumption that a smaller reaction scale is more favorable, whereby the performance of both steps on the 1 g scale gives the highest yield (entries 7 and 8). Regarding the product distribution for phenols **17a**–**d**, the two “mixed” experiments are in between the patterns observed for the sequences conducted on a “pure” 1 and 2 g scale. On this experimental basis, and as no variations in the used chemicals are obvious, we currently assign the deviations between the reaction scales—concerning both yield and product distribution—to technical differences associated with the reaction scale, e.g., the slightly slower heating of a reaction conducted on a larger scale. Although such deviations in the product pattern might, on the one hand, appear as a negative aspect, they can ultimately also turn out as useful, since depending on the conditions, one could possibly target the sequence at one or two phenols as preferred products.

**Scheme 4:**
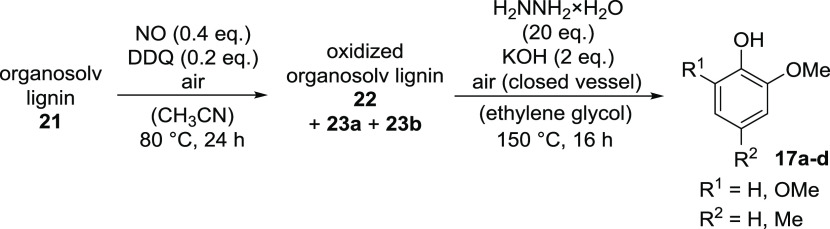
Optimized Conditions for the Two-Step Sequence Converting Organosolv Lignin (**21**) into Phenols **17a**–**d**

Taking into account the results from Figures 2 and 4, the so far available optimized conditions for the two-step sequence are as shown in Scheme 4. A calculation of the *E* factor and the process mass intensity (PMI) for the currently optimized conditions (see the Methods section for precise amounts and the Supporting Information for calculation) led to a value of *E* = 102 (PMI = 103).^27^ This value is not as good, but still compares reasonably well with the *E* factor of 70 (PMI = 71) determined for the two-step process by Westwood,^12d^ which serves as a reference point for the present study.

In the next part, we turned to a further comparison of the newly established reaction conditions with those previously reported by the Westwood group,^12d^ as it appeared possible that the formation of phenols **17a**–**d** was also caused by the particular nature of the used organosolv lignin. If that was true, conditions such as those reported by the Westwood group,^12d^ might then also produce phenols of type **17**, although these conditions had originally been reported to give ketones **8** and **11** (Figure 1). The results obtained from the comparison and partial replacement of the conditions for the two reaction steps are summarized in Table 1, whereby all reactions were carried out on a 1 g scale.^12d^

**Table 1:**
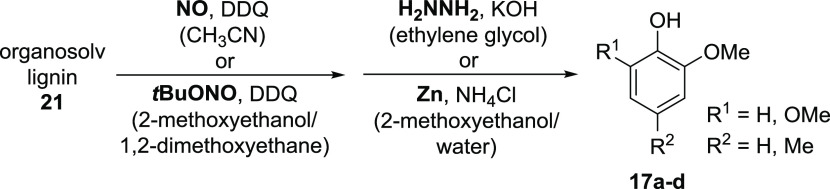
Comparison of Reaction Conditions in the Two-Step Sequence

entry	oxidation	reduction	**17a** (%)	**17b** (%)	**17c** (%)	**17d** (%)	**17a**–**d** (%)
1	***t*****BuONO**/DDQ	**Zn**/NH_4_Cl^a^	0.0	0.0	0.0	0.0	0.0
2	***t*****BuONO**/DDQ	**H**_**2**_**NNH**_**2**_/KOH	0.1	0.09	0.4	0.52	**1.11**
3	**NO**/DDQ	**Zn**/NH_4_Cl	0.0	0.0	0.0	0.0	0.0
4	**NO**/DDQ	**H**_**2**_**NNH**_**2**_/KOH	2.08	2.36	1.67	0.79	**6.9**
			1.51	2.05	1.07	0.74	**5.4**

a Reactions performed on a 1 g scale. Yields determined after purification by column chromatography using maleic acid as internal standard. Yields over two steps based on organosolv lignin **21**.

The observation that the Westwood sequence^12d^ involving *tert*-butyl nitrite and zinc (entry 1) does not lead to phenols **17a**–**d** suggests that phenol formation is probably due to the particular cleavage conditions and not by the type of lignin that is used. Instead, the formation of the originally reported ketones **8** and **11**(12d) could be confirmed by the analysis of the crude reaction mixture (entry 1).

**Figure 5:**
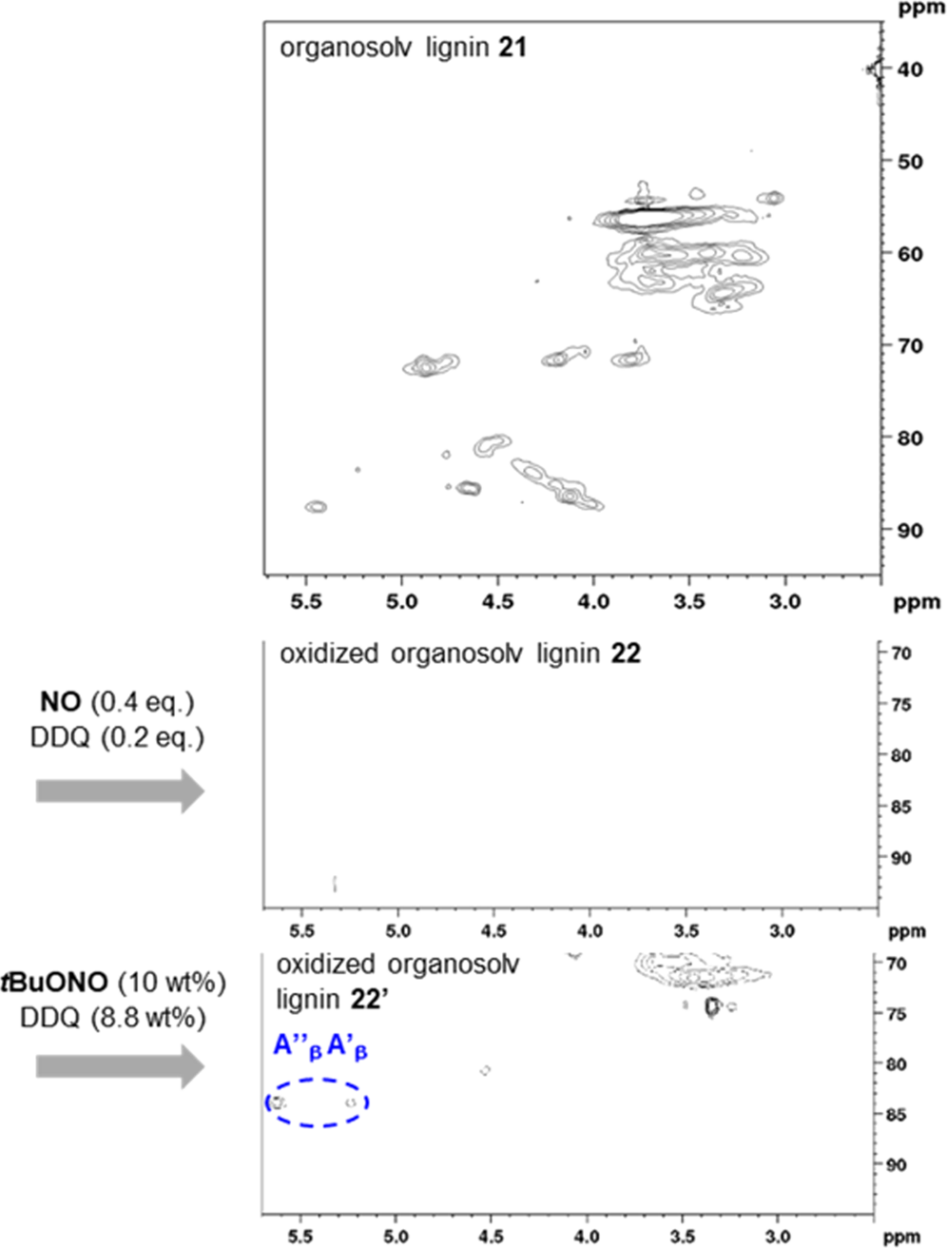
Comparison of the oxidation of organosolv lignin **21** by nitrogen monoxide/DDQ and *tert*-butyl nitrite/DDQ on the 1 g scale.

Regarding entries 2 and 3, the decisive step to direct the two-step sequence toward phenolic products appears to be the hydrazine-mediated reduction, as no detectable yields of **17a**–**d** were observed when the oxidation by nitrogen monoxide was combined with a zinc-mediated reduction. From all entries 1–4, one can conclude that the so far best sequence to obtain phenols **17** is that reported herein, namely, the combination of nitrogen monoxide with hydrazine. Pyrazoles **20**, as they were found as cleavage products in the reactions with the lignin systems **18** (Scheme 1), were not detected in any of the four experiments summarized in Table 1. Support for the diverse experimental results in Table 1 could be obtained from 2D NMR spectra, which show that the DDQ-catalyzed oxidation of organosolv lignin **21** with *tert*-butyl nitrite indeed leads to a differently oxidized lignin **22′** compared to **22** obtained with nitrogen monoxide (Figure 5).

The most striking difference are the cross-peaks for A_β_′ and A_β_″, which can unambiguously be assigned to precise structural fragments **A′** and **A″** depicted in Figure 3. These signals are present in the HSQC spectrum of oxidized lignin **22′**, but not in the one of **22**.

The four combinations of reaction conditions shown in Table 1 were also applied to air-dried Birch lignin (see the Supporting Information). This type of lignin gave a comparable result under the conditions combining *tert*-butyl nitrite and hydrazine (Table 1, entry 2), and provided phenols **17a**–**d** in 1.08% total yield, but turned out as far less well suited under the optimal conditions comprising nitrogen monoxide and hydrazine (Table 1, entry 4), as only 0.87% total yield was reached. Good results were achieved with an advanced model for birch lignin prepared according to a procedure previously established by the Westwood group,^28^ as this sample led to phenols **17a** (1.67%), **17b** (1.75%) **17c** (1.25%), and **17d** (0.5%) with a comparably good total yield of 5.17% after treatment under the optimized conditions of Scheme 4 comprising nitrogen monoxide and hydrazine. Although the overall yield may benefit to some extent from the smaller scale (0.5 g), this experiment represents an important connection between the lignin systems **18**(24) (Scheme 1) and the organosolv lignin **21** mainly used as starting material in the present work. Regarding the results obtained with the advanced model, it becomes obvious that the major changes in outcome occur when turning from the simple lignin systems **18** (Scheme 1) to the advanced model, and by far not as much deviation is observed between the advanced model and the organosolv lignin **21**. In turn, it is the further functionalization of the aromatic residues in the simple lignin systems **18** by alkyl residues, as present in fragment **A** (Figure 3), that not only leads to a far lower yield of phenols as cleavage products but also to the suppression of pyrazole formation.

**Scheme 5:**
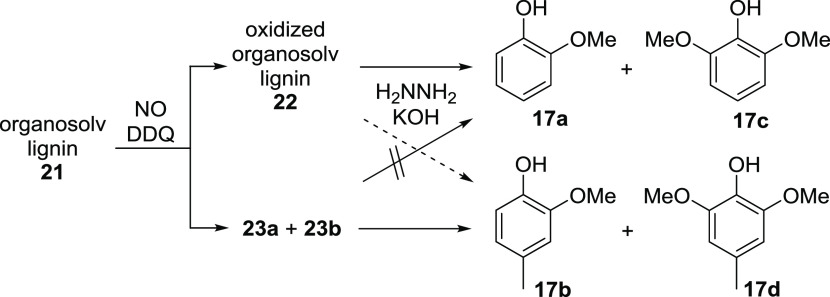
Formation of Phenols **17a**–**d** from Organosolv Lignin **21**

Regarding plausible pathways leading to the formation of phenols **17a**–**d** from organosolv lignin (Scheme 5), the intermediates vanillin (**23a**) and syringaldehyde (**23b**) are well known as possible cleavage products resulting from lignin under oxidative conditions (Figure 1).^7a,7b,9a,9b,12b,26^

Control experiments revealed that **23a** and **23b** are even present in small amounts in the organosolv lignin **21** used as starting material (**23a**: 1.11%, **23b**: 0.53%), but when treated separately under the oxidative conditions comprising nitrogen monoxide and DDQ, they turned out to be unstable, and full decomposition to a complex product mixture was observed. Accordingly, the aldehydes **23a** and **23b** are formed as well as partially decomposed in the oxidation step, leading from **21** to oxidized lignin **22**, which is a plausible background for deviations in yields even in identical experiments.

Concerning the reductive step, and regarding literature, vanillin (**23a**) has been successfully converted to **17b** using hydrazine and potassium hydroxide at elevated temperatures,^29^ which would also explain the occurrence of the second ring-methylated phenol **17d**. The assumption that the aldehydes **23a** and **23b** are direct precursors of the 4-methylphenols **17b** and **17d** was confirmed by reacting both aldehydes with hydrazine under the optimized reductive conditions to give **17b** and **17d** in yields of 57 and 41%, respectively. In agreement with this is also the observation that when the reduction step is carried out under “open” instead of “closed” conditions so that the reductant hydrazine is likely to be consumed faster to nitrogen due to its facilitated access to oxygen,^30^ the yield of phenol **17b**, and in most cases also that of **17d**, decreases (Figure 4).

The fact that phenols **17a** and **17c** were not obtained in the hydrazine-mediated reductions of **23a** and **23b** necessitates their formation from the oxidized organosolv lignin **22**. On the other hand, we can currently not exclude that additional amounts of **17b** and **17d** arise from **22** upon reduction.

Finally, it was shown that the direct submission of organosolv lignin **21** to the reductive cleavage conditions comprising hydrazine and potassium hydroxide does not lead to detectable amounts of phenols **17a**–**d**, thereby indicating that the small amounts of **23a** and **23b** being present in the starting material **21** are insufficient to enable a direct access to **17b** and **17d** through reduction.

Besides these considerations, the pathway for the formation of phenols **17a** and **17c** is yet difficult to explain. A known reaction leading to the cleavage of an aryl–alkyl C–C bond located at electron-rich aromatics is the *ipso*-nitration reported by Bozell,^31^ where under the conditions comprising nitrous acid in the presence of oxygen would be very well comparable with the oxidative conditions employed in this work. However, we were unable to detect the related nitrophenols along with the oxidized lignin **22** and the aldehydes **23a** and **23b**, which could have pointed to this pathway.

## Conclusions

In summary, it has been shown that phenols can be obtained from organosolv lignin by a two-step sequence comprising DDQ-catalyzed oxidation with nitrogen monoxide followed by hydrazine-mediated cleavage and reduction. Under the so far optimized conditions, the phenols **17a**–**d** were formed in a total yield of up to 6.9% (4.9 wt %) over the two reaction steps. In addition, the reaction scale was shown to have an influence on both yield and product distribution. While the formation of the two 4-methylphenols **17b** and **17d** can be explained by the intermediate occurrence of vanillin (**23a**) and syringaldehyde (**23b**) after the oxidation step, the mechanistic pathways leading to 2-methoxyphenol (**17a**) and 2,6-dimethoxyphenol (**17c**) remain to be investigated. For the formation of these compounds, a C–C bond adjacent to an electron-rich aromatic system has to be reductively cleaved to an aryl C–H bond, which has—to the best of our knowledge—not yet been reported in the context of lignin depolymerization. Phenols that are unsubstituted in the 4-position or bear a 4-methyl group, such as compounds **17a**–**d**, do typically not occur as major products in known strategies comprising an oxidative and a reductive substep.^12c−12e,13^ However, such phenols can be obtained by mechanochemical oxidation^15^ and high-temperature alkaline depolymerization.^16^ Herein, and besides the fact that two waste materials—namely, nitrogen monoxide and lignin—were combined in a metal-free valorization process, we have further shown that untypical forms of oxidized organosolv lignin such as **22** may be a key to further broaden the spectrum of depolymerization products.

## Methods

### Oxidation

To a solution of organosolv lignin (1.0 g, 5.0 mmol, 1.0 equiv) in acetonitrile (60 mL) in a round-bottom flask with a reflux condenser and a balloon for pressure compensation on top, DDQ (227 mg, 1.0 mmol, 0.20 equiv) was added and the mixture was heated up to 80 °C. In the meantime, nitrogen monoxide was synthesized according to the general procedure for nitrogen monoxide production. At 80 °C, nitrogen monoxide (44.8 mL, 2.0 mmol, 0.40 equiv) was added to the reaction via a syringe. The reaction mixture was stirred for 24 h at 80 °C. Afterward, the solvent was removed under reduced pressure and the oxidized lignin was further used without processing.

### Reduction

Oxidized organosolv lignin **22** (further containing **23a** and **23b**) (1.0 g, 5.0 mmol, 1.0 equiv), hydrazine monohydrate (4.9 mL, 100 mmol, 20 equiv), and potassium hydroxide (0.56 g, 10 mmol, 2.0 equiv) were dissolved in ethylene glycol (10 mL), and the mixture was stirred for 16 h at 150 °C under air in a closed reaction vessel with a balloon on top for pressure equilibration. Afterward, the reaction mixture was diluted with water (10 mL) and extracted with ethyl acetate at neutral (pH = 7) and acidic (pH = 3–4) pH. The pH value was adjusted with 5 M HCl. The combined organic phases were washed with saturated sodium chloride solution, dried over sodium sulfate, and the solvent was removed under reduced pressure not lower than 200 mbar due to the volatility of the phenolic products. After flash column chromatography (hexane/ethyl acetate = 4:1), the yields of the products **17a** (2.08%), **17b** (2.36%), **17c** (1.67%), and **17d** (0.79%) were determined by ^1^H NMR spectroscopy using maleic acid as internal standard.

## Supporting Information Available

Further experimental procedures, characterization of compounds, and NMR spectra (PDF)

## Abbreviations

DDQ: 2,3-dichloro-5,6-dicyano-1,4-benzoquinone
HSQC: heteronuclear single quantum coherence spectroscopy
NMR: nuclear magnetic resonance
TEMPO: 2,2,6,6-tetramethylpiperidin-1-yl)oxyl
