# Supplementary material for: Cleavage of Organosolv Lignin to Phenols Using Nitrogen Monoxide and Hydrazine
Source: ACS Omega. 2021 Jul 23;6(30):19400–8. doi: 10.1021/acsomega.1c00996 (PMC8340100; doi:10.1021/acsomega.1c00996)
Supplement: Supplementary file 1 — ao1c00996_si_001.pdf [file ao1c00996_si_001.pdf]

Supporting Information

Cleavage of organosolv lignin to phenols using nitrogen monoxide and hydrazine

Laura Elena Hofmann,[a] Lisa-Marie Altmann,[a] Oliver Fischer,[a] Lea Prusko,[a] Ganyuan Xiao,[b] Nicholas J. Westwood,[b] Markus R. Heinrich[a,]*

[a]Department of Chemistry and Pharmacy, Pharmaceutical Chemistry, Friedrich-Alexander-Universität Erlangen-Nürnberg, Nikolaus-Fiebiger-Straße 10, 91058 Erlangen, Germany

markus.heinrich@fau.de

[b]School of Chemistry and Biomedical Sciences Research Complex, University of St Andrews and EaStCHEM, North Haugh, St Andrews, Fife, United Kingdom

Table of Contents

1. General Remarks 2
2. Oxidation of organosolv lignin 5
   2.1 General procedure for oxidation 5
   2.2 General procedure for nitrogen monoxide production[2] 5
   2.3 2D HSQC NMR spectra of organosolv lignin and oxidized samples (Figure 2) 7
3. Cleavage of oxidized organosolv lignin 9
   3.1 General procedure for experimental data summarized in Figure 4 9
   3.2 Optimization of cleavage conditions (data in Figure 4) 9
   3.3 Combination of reaction conditions with those developed by *Westwood et al.*[4] 11
   3.4 Synthesis of the advanced model for birch lignin 13
4. Control reactions with the oxidation products vanillin (**23a**) and syringaldehyde (**23b**) 15
5. Extraction of air-dried birch lignin[4] 17
6. Characterization of oxidation and cleavage products 18
7. NMR spectra 21
8. References 37

1. General Remarks

Solvents and reagents were obtained from commercial sources and were used as received. NMR spectra were recorded on *Bruker* Avance 600 (^{1}H: 600 MHz, ^{13}C: 151 MHz) and *Bruker* Avance 400 (^{1}H: 400 MHz, ^{13}C: 101 MHz). For ^{1}H NMR $CDCl_3$, DMSO-d_6 was used as solvent referenced to TMS (0.00 ppm), $CDCl_3$ (7.26 ppm) and DMSO-d_6 (2.50 ppm). For DEPTQ and ^{13}C NMR DMSO-d_6 referenced to DMSO-d_6 (39.52 ppm) was used as solvent. For 2D HSQC NMR spectra DMSO-d_6 referenced to DMSO-d_6 (^{1}H: 2.50 ppm, ^{13}C: 39.52 ppm) was used. Chemical shifts are reported in parts per million (ppm). Coupling constants are in Hertz (*J* Hz). The following abbreviations are used for the description of signals: s (singlet), d (doublet), t (triplet), q (quartet), m (multiplet) and bs (broad singlet).

Analytical TLC was carried out on *Merck* silica gel plates using short wave (254 nm) UV light and CAM [1.0 g $Ce(SO_4)_2$, 29 g ammonium molybdate, 25 mL H_2SO_4 in 200 mL H_2O] to visualize components.

Silica gel (Kieselgel 60, grain size 40-63 µm, *Merck*) was used for column chromatography.

Organosolv lignin was generously provided by the Fraunhofer-Zentrum für Chemisch-Biotechnologische Prozesse CBP, Leuna.[1]

The yields were calculated on the basis of the mass of the organosolv lignin used with a rounded average mass of 200 g/mol per monomer. According to this, a 1 g experiment (= mass of organosolv lignin) corresponds to 5 mmol of lignin monomers, and the amount of 5 mmol was used as basis (= 100%) for the calculation of the yields of the finally obtained phenols. Also the amounts of reagents used were calculated on these 5 mmol (as an example).

Calculation of the S:G ratio

The ratio of sinapyl to coniferyl monomers (S:G ratio) in the starting material (organosolv lignin) was calculated from 2D NMR spectra (see Figure 1, below) as follows: S:G = (1:2): [(0.14+0.30+0.11)/3] = 2.73:1.

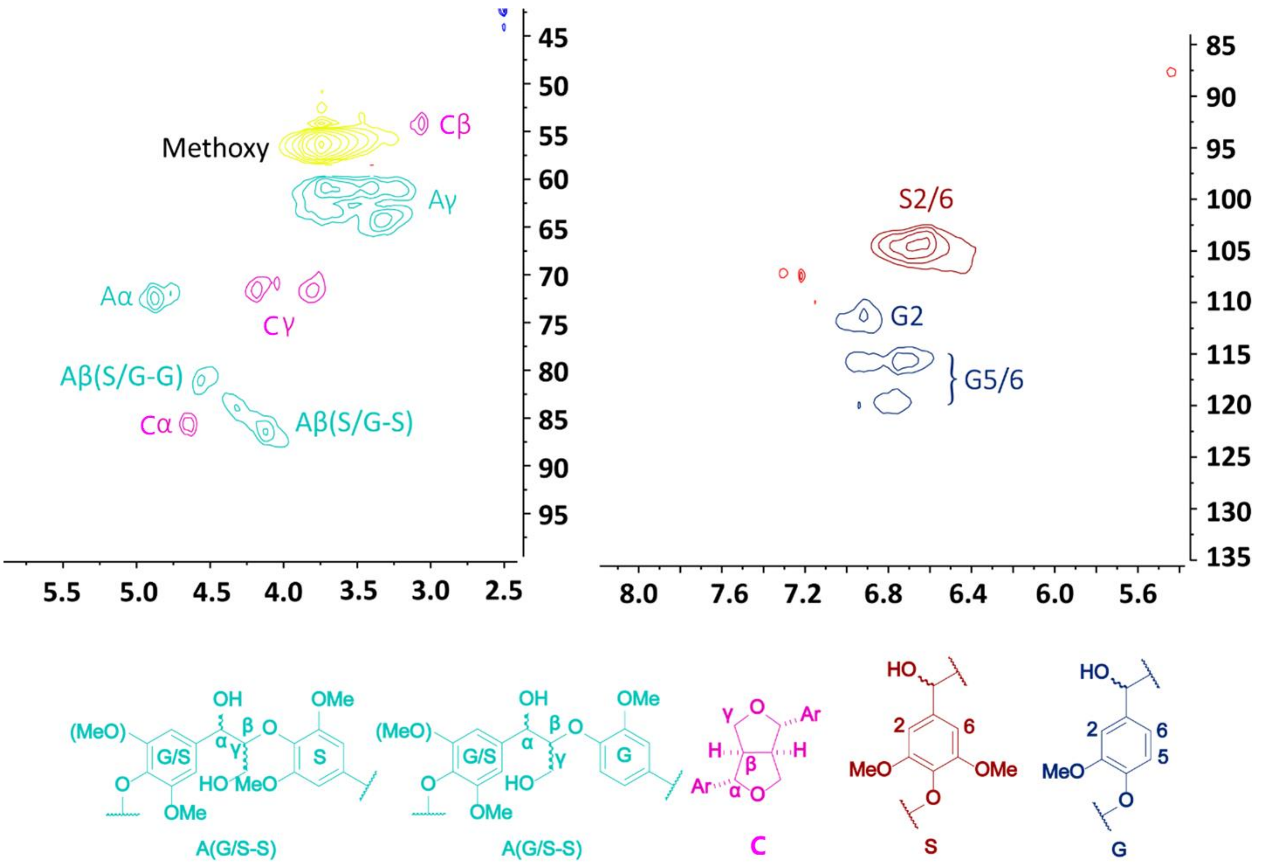

Figure S1. 2D NMR spectra for the calculation of the ratio of sinapyl to coniferyl monomers (S:G ratio) in the starting material.

Calculation of number of β-O-4, β-β and β-5 units in organosolv lignin 21

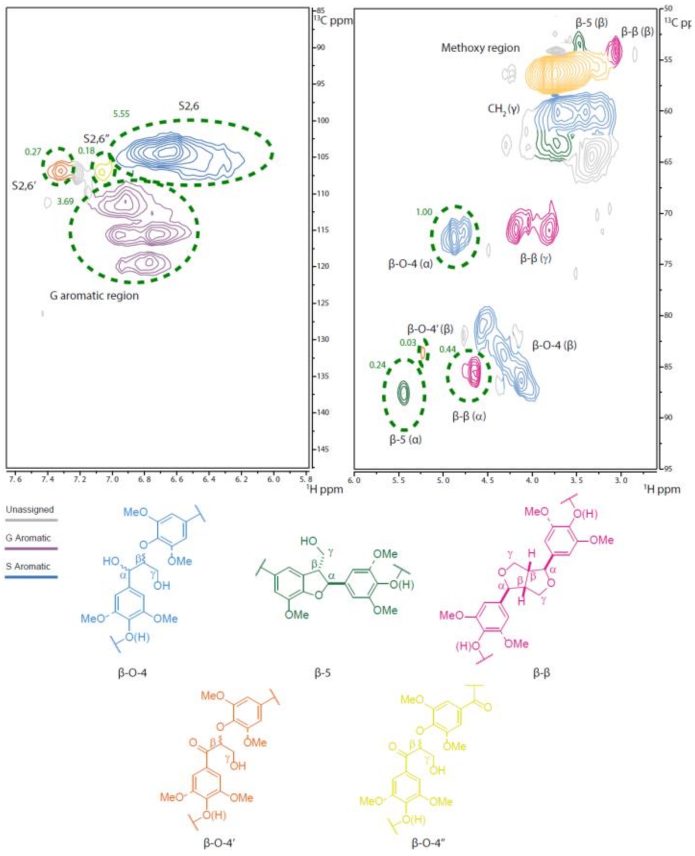

Figure S2. Analysis by 2D HSQC NMR spectra for the calculation of the number of β-O-4, β-β and β-5 units per 100 C9 units in the starting organosolv lignin **21**.

The α-proton of the β-O-4 unit has been set to an integral value of 1.00

$S_{2,6}$ = 5.55 (2H)

$S_{2,6'}$ = 0.18 (2H) – oxidised β-O-4' units in starting organosolv lignin **21**

$S_{2,6''}$ = 0.27 (2H) – oxidised β-O-4'' units in starting organosolv lignin **21**

$S_{condensed}$ = 0

$G_{2,4,5}$ = 3.69 (3H)

Total C9 units = (5.55/2) + (0.18/2) + (0.28/2) + 0 + (3.69/3) = 4.235 per β-O-4 unit

Number of β-O-4 units per C_9 unit = 1.00/4.235 = 0.24 therefore 24 β-O-4 units per 100 C_9 units

Number of β-β units per C_9 unit = (0.44/2)/4.235 = 0.052 therefore 5 β-β units per 100 C_9 units

Number of β-5 units per C_9 unit = (0.24)/4.235 = 0.057 therefore 6 β-5 units per 100 C_9 units

An additional calculation of the S:G ratio based on Figure 2 gave a ratio of 2.44:1 which is in good agreement with the ratio of 2.73:1 calculated on the basis of Figure 1.

2. Oxidation of organosolv lignin

2.1 General procedure for oxidation

To a solution of organosolv lignin (M = ø 200 g/mol) in acetonitrile (60 ml/g) in a round bottom flask with a reflux condenser and a balloon for pressure compensation on top, DDQ was added and the mixture was heated up to 80 °C. In the meantime, nitrogen monoxide was synthesized according general procedure for nitrogen monoxide production. At 80 °C, nitrogen monoxide was added to the reaction *via* a syringe. The reaction mixture was stirred for 18-24 h at 80°C. Afterwards, the solvent was removed under reduced pressure and the oxidized lignin was further used without processing. For detailed reaction conditions, e.g. the amounts of organosolv lignin and DDQ, see Figure 2 in the article.

2.2 General procedure for nitrogen monoxide production[2]

Nitrogen monoxide was synthesized using sodium nitrite (6.8 g) and potassium iodide (2.6 g) in a 500 mL three-necked flask. A funnel was plugged into the middle neck of the flask and the connection between flask and funnel was closed airtight. One of the other two necks was also closed with a septum. 1M H_2SO_4 was filled into the flask until it was free from air, sodium nitrite was added, and the mixture was stirred. The third neck was then closed with a septum (see Figure S3 of empty setup (left) below). Potassium iodide was dissolved in water (10 mL) and slowly added to the flask through one of the septums *via* a syringe. Through the developing nitrogen monoxide, the aqueous phase was partially pushed up into the funnel. For oxidation reactions, nitrogen monoxide was taken *via* a syringe out of the gas phase in the flask and added to the reaction.

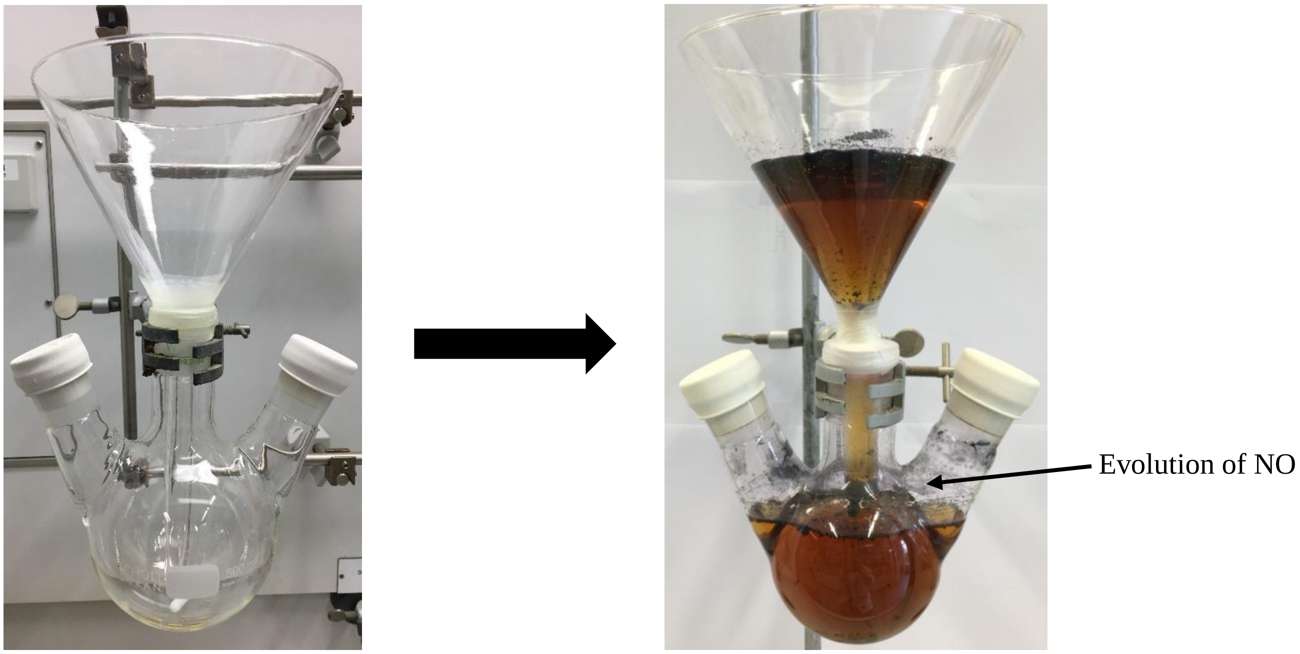

Figure S3. Experimental setup for the synthesis of nitrogen monoxide comparable to Kipps' apparatus.

2.3 2D HSQC NMR spectra of organosolv lignin and oxidized samples

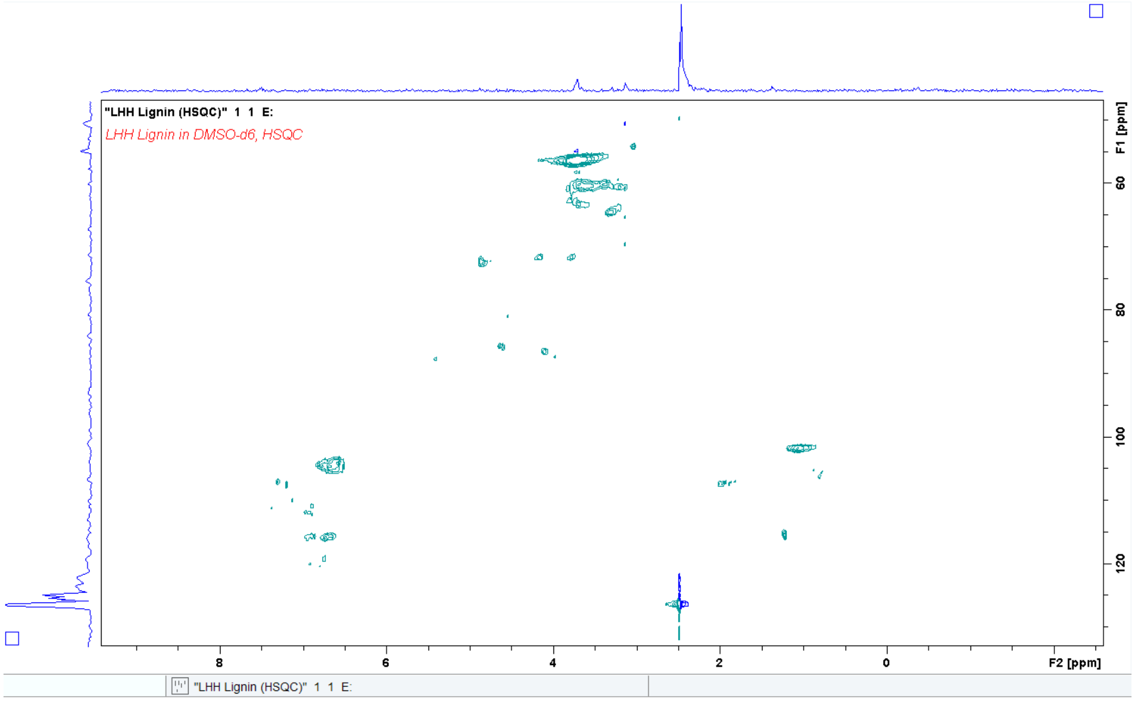

Figure S4. Full 2D HSQC NMR spectra of organosolv lignin **21** (Figure 2, **I**) – low threshold level set when image was generated. This spectrum was obtained using a restricted 13C region resulting in folding of the aliphatic signals.

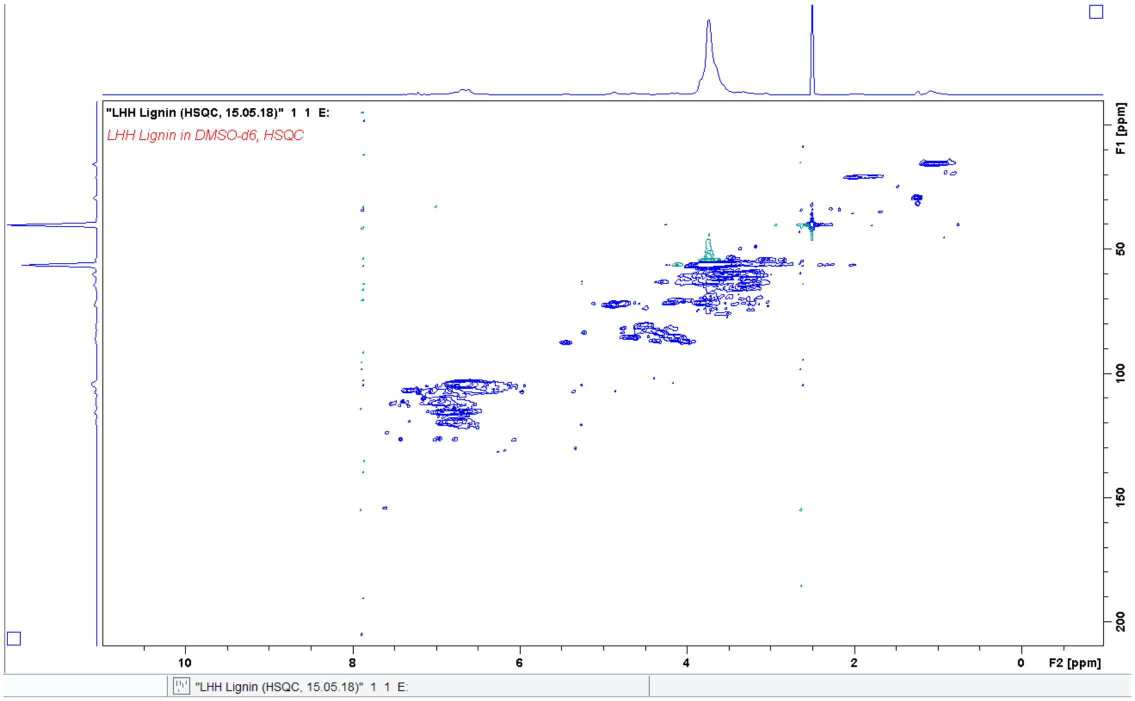

Figure S5. Full 2D HSQC NMR spectra of organosolv lignin **21** (Figure 2, **I**, increased threshold level compared to Figure S3).

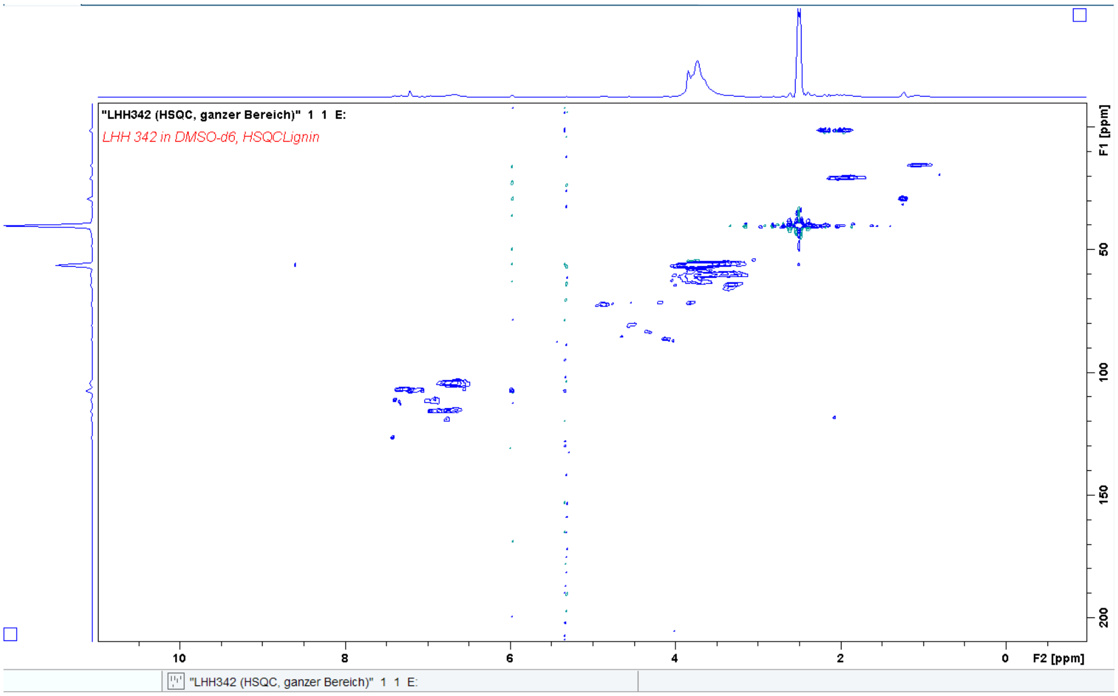

Figure S6. Full 2D HSQC NMR spectrum of oxidized organosolv lignin **22** (Figure 2, **II**)

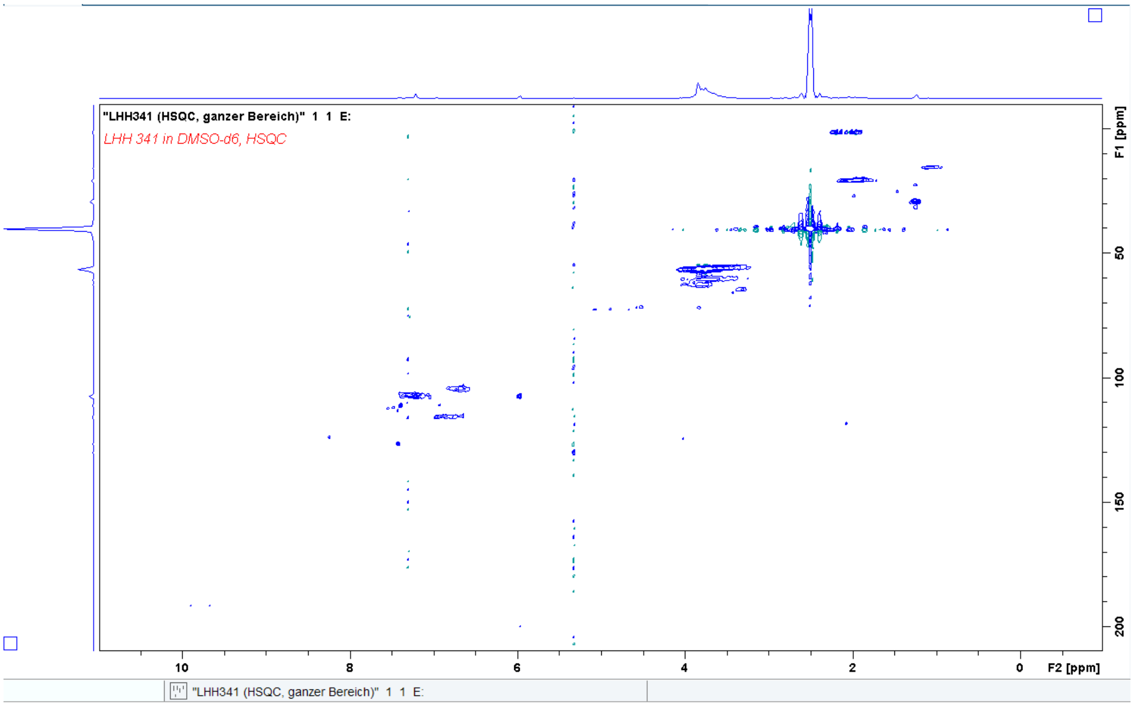

Figure S7. Full 2D HSQC NMR spectrum of oxidized organosolv lignin **22** (Figure 2, **III**)

3. Cleavage of oxidized organosolv lignin

3.1 General procedure for experimental data summarized in Figure 4

Oxidized organosolv lignin (for synthesis see above or see Scheme 2 in the article), hydrazine monohydrate and potassium hydroxide were dissolved in ethylene glycol in a round-bottom flask with a reflux condenser on top and the mixture was stirred for 16 h at 150 °C under air either under open or closed reaction conditions. For closed reaction conditions the reflux condenser was closed with a septum and a balloon on top for pressure compensation. Afterwards the reaction mixture was diluted with water and extracted with ethyl acetate neutral (pH=7) and acidic (pH=3-4). The pH value was adjusted with 5 M HCl. The combined organic phases were washed with saturated sodium chloride solution, dried over sodium sulfate and the solvent was removed under reduced pressure not lower than 200 mbar. After flash column chromatography (hexane : ethyl acetate = 4:1), the yields were determined by ^{1}H NMR spectroscopy using maleic acid as internal standard. For detailed reaction conditions and yields see Tables S1, S2 and S3.

3.2 Optimization of cleavage conditions (data in Figure 4)

Table S1. Optimization reactions on 1 g scale.

entry (open/closed)	hydrazine [equiv]	KOH [equiv]	**17a** [%]	**17b** [%]	**17c** [%]	**17d** [%]	**17a-d** [%]
1 (open)	20	-	1.7	-	2	-	3.7
2 (open)	20	-	1.7	0.55	1.8	-	4.05
3 (open)	20	0.5	1.2	-	1.6	0.4	3.2
4 (open)	10	2	0.8	-	2.6	-	3.4
5 (open)	20	2	1.08	0.4	2.25	1.2	4.93
6 (open)	20	2	1.12	-	1.8	1.1	4.02
7 (closed)	20	2	2.08	2.36	1.67	0.79	6.9
8 (closed)	20	2	1.51	2.05	1.07	0.74	5.4

General procedure: oxidized organosolv lignin (**22**) (1.0 equiv), KOH (0-2.0 equiv), H_2NNH_2 (10-20 equiv), ethylene glycol (10 mL), 150 °C, 16 h in an air-filled either open or closed reaction vessel. Yields determined after flash column chromatography by ^{1}H-NMR using maleic acid as internal standard. Yields are based on lignin.

Table S2. Optimization reactions on 2 g scale.

entry (open/closed)	hydrazine [equiv]	KOH [equiv]	**17a** [%]	**17b** [%]	**17c** [%]	**17d** [%]	**17a-d** [%]
9 (open)	20	-	0.23	-	2.4	-	2.63
10 (open)	10	2	0.34	0.45	1.07	0.63	2.49
11 (open)	20	2	0.3	0.27	1.1	0.33	1.51
12 (closed)	20	2	0.47	0.63	1.2	0.86	3.16
13 (closed)	20	2	0.4	0.58	1.1	0.75	2.83

General procedure: oxidized organosolv lignin (**22**) (1.0 equiv), KOH (0-2.0 equiv), H_2NNH_2 (10-20 equiv), ethylene glycol (20 mL), 150 °C, 16 h in an air-filled either open or closed reaction vessel. Yields determined after flash column chromatography by ^{1}H-NMR spectroscopy using maleic acid as internal standard. Yields are based on lignin.

Table S3. Mixed reactions.

entry	hydrazine [equiv]	KOH [equiv]	**17a** [%]	**17b** [%]	**17c** [%]	**17d** [%]	**17a-d** [%]
14 (closed)	20	2	0.86	0.53	1.5	0.38	3.27
15 (closed)	20	2	0.45	1.2	1.4	0.4	3.45

General procedure: oxidized organosolv lignin (**22**) (1.0 equiv), KOH (2.0 equiv), H_2NNH_2 (20 equiv), ethylene glycol (10-20 mL), 150 °C, 16 h in an air-filled closed reaction vessel. Yields determined after flash column chromatography by ^{1}H-NMR spectroscopy using maleic acid as internal standard. Yields based on lignin.

Calculation of E factor and process mass intensity (PMI)

The E factor and the process mass intensity were calculated as shown in Figure S8 according to ref.[3] Solvents are not included in the calculation as they are recoverable.

$$E = \frac{\text{mass of raw materials - mass of products } \mathbf{17a\text{-}d}}{\text{mass of products } \mathbf{17a\text{-}d}} \qquad PMI = \frac{\text{total mass in process}}{\text{mass of products } \mathbf{17a\text{-}d}} = E + 1$$

$$E = \frac{\overset{\text{lignin}}{1000} + \overset{\text{DDQ}}{227} + \overset{\text{NO}}{60} + \overset{N_2H_4}{3200} + \overset{\text{KOH}}{560} - \overset{\text{products}}{49}}{49} = 102 \qquad PMI = 103$$

values for 2-step process (in mg)

Figure S8. Calculation of E factor and PMI using the optimized conditions (see experimental section of the manuscript)

For comparison, the E factor and the PMI were also calculated for the related two-step process by *Westwood*[4] (Figure S9).

$$E = \frac{\overset{\text{lignin}}{2400} + \overset{\text{DDQ}}{240} + \overset{\text{TBN}}{240} + \overset{NH_4Cl}{3000} + \overset{\text{Zn}}{3600} - \overset{\text{products}}{133}}{133} = 70 \qquad PMI = 71$$

values for 2-step process (in mg)

Figure S9. Calculation of E factor and PMI for the two-step procedure by Westwood[4] using TBN/DDQ for oxidation and Zn for reductive cleavage (see Scheme 3 in ref. 4).

3.3 Combination of reaction conditions with those developed by *Westwood et al.*[4]

Procedure for organosolv lignin oxidation with TBN/DDQ

In a round-bottom flask organosolv lignin (**21**) (1.0 g), DDQ (100 mg, 10 wt%) and TBN (102 µL, 8.8 wt%) were dissolved in 2-methoxyethanol/1,2-dimethoxyethane (2:3, 14.0 mL) and placed under O_2 atmosphere. The reaction was heated to 80 °C and stirred for 14 h. The oxidized lignin was precipitated in 10 volumes of diethyl ether, filtered off and dried under vacuum.

Procedure for organosolv lignin oxidation with NO/DDQ

To a solution of **21** (1.0 g, 1.0 equiv) in acetonitrile (60 ml) in a round bottom flask with a reflux condenser and a balloon for pressure compensation on top of it, DDQ (227 mg, 0.20 equiv) was added and the mixture was heated up to 80 °C. In the meantime, nitrogen monoxide was synthesized according general procedure for nitrogen monoxide production. At 80 °C self-synthesized nitrogen monoxide (44.8 mL, 0.40 equiv) was added to the reaction *via* a syringe. The

reaction was stirred for 24 h. Afterwards, the solvent was removed under reduced pressure and the oxidized organosolv lignin was further used without any processing.

Procedure for zinc-mediated cleavage of oxidized organosolv lignin (Table 1, entries 1 and 3)

Oxidized organosolv lignin (**22**) (600 mg), either oxidized by NO/DDQ (entry 3) or TBN/DDQ (entry 1), was dissolved in 2-methoxyethanol (8.40 mL) and water (2.10 mL). NH_4Cl (740 mg) and Zn (900 mg) were added and the mixture was heated up to 80 °C and stirred for 1 h. Afterwards, the reaction mixture was allowed to cool, was filtered to remove excess of Zn, and water (30 mL) was added. The mixture was acidified to pH 1 by 1M HCl causing lignin to flocculate and it was filtered again. Residual lignin was washed with ethyl acetate and the aqueous filtrate was extracted with ethyl acetate (5 × 20 mL). The combined organic phases were washed with saturated $NaHCO_3$, sodium chloride, dried over sodium sulfate and concentrated under reduced pressure not lower than 200 mbar. For entry 1 (oxidized by TBN/DDQ), the crude product was purified by flash column chromatography (hexane : ethyl acetate = 1:1 → ethyl acetate → ethyl acetate + 1% methanol). No phenols **17a-d** could be detected after flash column chromatography. For entry 3 (oxidized by NO/DDQ), the crude product was purified by flash column chromatography (hexane : ethyl acetate = 4:1) and no phenols **17a-d** could be detected.

Procedure for hydrazine-induced cleavage of oxidized organosolv lignin (Table 1, entry 2)

By TBN/DDQ oxidized organosolv lignin (**22**) (965 mg, 1.0 equiv), hydrazine monohydrate (4.73 mL, 20.0 equiv) and potassium hydroxide (544 mg, 2.00 equiv) were dissolved in ethylene glycol (10 mL) and the mixture was stirred for 16 h at 150 °C under air in a closed reaction vessel with a balloon on top for pressure compensation. Afterwards, the reaction mixture was diluted with water (10 mL) and extracted with ethyl acetate neutral (pH=7) and acidic (pH=3-4). The pH value was adjusted with 5M HCl. The combined organic phases were washed with saturated sodium chloride solution, dried over sodium sulfate and the solvent was removed under reduced pressure not lower than 200 mbar. After flash column chromatography (hexane : ethyl acetate = 4:1), the yields of the products **17a** (0.1%), **17b** (0.09%), **17c** (0.4%) and **17d** (0.52%) were determined by ^{1}H-NMR spectroscopy using maleic acid as internal standard.

Procedure for hydrazine-induced cleavage of oxidized organosolv lignin (Table 1, entry 4)

By NO/DDQ oxidized organosolv lignin (**22**) (1.20 g, 1.0 equiv), hydrazine monohydrate (5.90 mL, 20.0 equiv) and potassium hydroxide (680 mg, 2.00 equiv) were dissolved in ethylene glycol (10 mL) and the mixture was stirred for 16 h at 150 °C under air in a closed reaction vessel with a balloon on top for pressure compensation. Afterwards, the reaction mixture was diluted with water (10 mL) and extracted with ethyl acetate neutral (pH=7) and acidic (pH=3-4). The pH value was adjusted with 5M HCl. The organic phase was washed with saturated sodium chloride solution, dried over sodium sulfate and the solvent was removed under reduced pressure not lower than 200 mbar. After flash column chromatography (hexane : ethyl acetate = 4:1), the yields of the products **17a** (2.08%), **17b** (2.36%), **17c** (1.67%) and **17d** (0.79%) were determined by ^{1}H-NMR spectroscopy using maleic acid as internal standard.

3.4 Synthesis of the advanced model for birch lignin

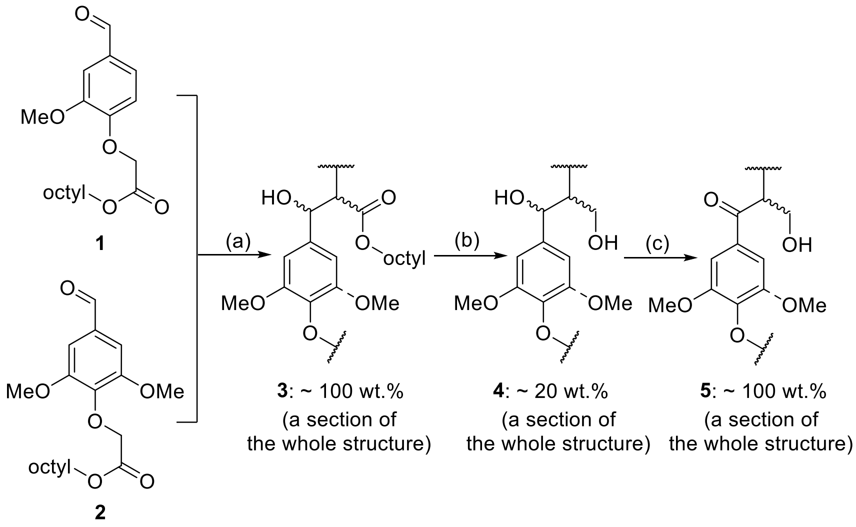

Scheme S1. The synthesis of oligomer **5**: (a) LDA (2.2 eq.), THF, -20 °C, 1.5 h, quant.; (b) $NaBH_4$ (5.0 eq.), MeOH (15.0 eq.), EtOH, 50 °C, overnight.; (c) DDQ (1 eq.), 1,4-dioxane/MeOH, 50 °C, overnight.

To a solution containing both octyl 2-(4-formyl-2-methoxyphenoxy)acetate (**1**, 10 g, 31 mmol, 0.5 eq.) and octyl 2-(4-formyl-2,6-dimethoxyphenoxy)acetate (**2**, 10.9 g, 31 mmol, 0.5 eq.) in THF (400 mL) was added dropwise a freshly prepared solution of LDA (74.4 mmol, 1.2 eq.) in THF

(60 mL) at -20 °C. The reaction mixture was stirred at -20 °C for 1.5 hour. Then the reaction was quenched by adding sat. NH_4Cl solution (100 mL). The reaction mixture was allowed to warm to room temperature. The reaction mixture was diluted with H_2O (100 mL) and ethyl acetate (200 mL). The phases were separated, and the aqueous layer was extracted with ethyl acetate (3 x 200 mL). The combined organic layers were washed with brine, dried over Na_2SO_4 and concentrated *in vacuo* to yield the crude polyester **3**. The crude product **3** was then dissolved in ethanol (300 mL). To this solution was added sodium borohydride (11.7 g, 310 mmol, 5 eq.) and the reaction mixture heated to 50 °C. Methanol (37.6 mL, 930 mmol, 15 eq.) was then added dropwise over 15 mins and the reaction maintained at 50 °C overnight. The reaction mixture was then concentrated *in vacuo* and the residue was taken up in water (400 mL) and the crude polymer precipitated by acidification with conc. HCl. The product was collected as a light-yellow gum and dried *in vacuo*. The crude polymer was then taken up in acetone/methanol (9:1, 30 mL), filtered and precipitated by dropwise addition to diethyl ether (400 mL). The polymer **4** was collected by filtration and dried *in vacuo* to give a white powder (4 g, ~20%). A section of the 2D HSQC analysis of **4** is shown in Figure S10 below.

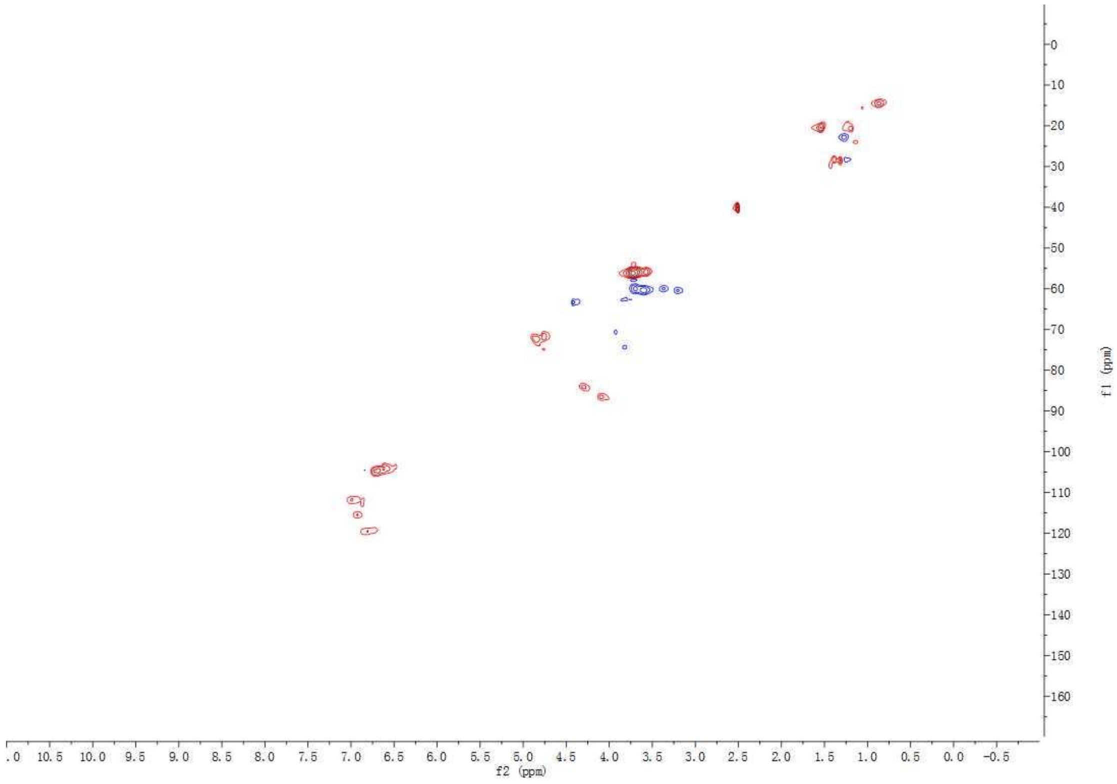

Figure S10. 2D HSQC NMR spectrum of polymer **4**.

The polymer **4** (500 mg) was dissolved in 1,4-dioxane/MeOH (9 mL/1 mL) and DDQ (600 mg) was added. The reaction was heated at 50 °C overnight. The reaction mixture was cooled to room temperature and precipitated into diether ether. The precipitated oxidized polymer **5** was collected by filtration and dried *in vacuo*. Yield of polymer **5**: ~ 100 wt%. A section of the 2D HSQC analysis of **5** is shown in Figure S11 below.

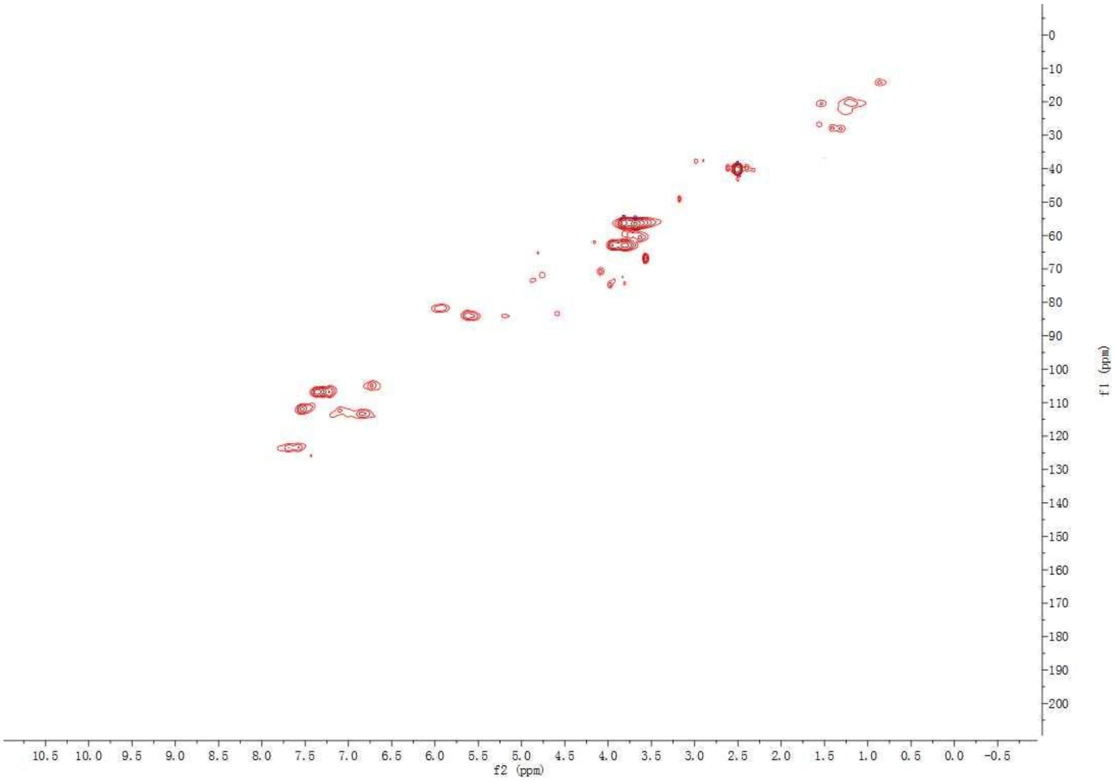

Figure S11. 2D HSQC NMR spectrum of polymer **5**.

4. Control reactions with the oxidation products vanillin (23a) and syringaldehyde (23b)

Control reactions A-B under oxidative conditions

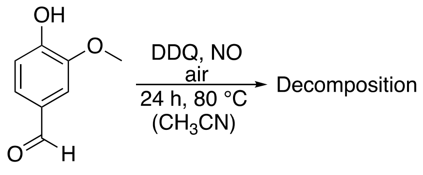

Scheme S2. Control reaction A.

Vanillin (**23a**, 0.632 mmol, 96 mg, 1.0 eq.) was treated with DDQ (1.0 mmol, 228 mg, 1.6 eq.) in acetonitrile (60 mL) at 80 °C according to the general NO/DDQ oxidation conditions. The reaction resulted in a complex mixture of unidentifiable substances. No traces of **23a** could be identified.

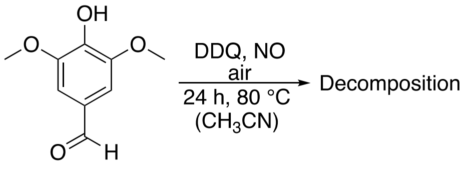

Scheme S3. Control reaction B.

Syringaldehyde (**23b**, 0.632 mmol, 115 mg, 1.0 eq.) was treated with DDQ (1.0 mmol, 228 mg, 1.6 eq.) in acetonitrile (60 mL) at 80 °C according to the general NO/DDQ oxidation conditions. The reaction resulted in a complex mixture of unidentifiable substances. No traces of **23b** could be identified.

Control reactions C-D under reductive cleavage conditions

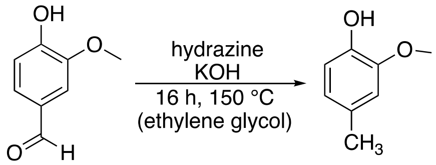

Scheme S4. Control reaction C.

Vanillin (**23a**, 0.632 mmol, 96 mg, 1.0 eq.) was treated with hydrazine monohydrate (253 mmol, 12.6 mg, 12.3 mL, 400 eq.) in ethylene glycol (20 mL) at 150 °C according to the general hydrazine-induced cleavage conditions. The crude product was analyzed using ^{1}H-NMR using ethylene carbonate as an internal standard. The phenol **17b** was found as a main product (0.36 mmol, 57% yield, 84% conversion).

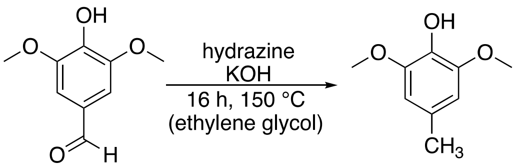

23b 17d

Scheme S5. Control reaction D.

Syringaldehyde (**23b**, 0.632 mmol, 115 mg, 1.0 eq.) was treated with hydrazine monohydrate (253 mmol, 12.6 mg, 12.3 mL, 400 eq.) in ethylene glycol (20 mL) at 150 °C according to the general hydrazine-induced cleavage conditions. The crude product was analyzed using ^{1}H-NMR using ethylene carbonate as an internal standard. The phenol **17d** was found as a main product (0.26 mmol, 41% yield, 67% conversion).

5. Extraction of air-dried birch lignin[4]

Air-dried birch sawdust (200.0 g) was mixed with 1,4-dioxane (1.44 L) and 2N HCl (160.0 mL), placed under N_2, heated to a gentle reflux and stirred for 1 h. The reaction mixture was allowed to cool, the liquor was collected by filtration and concentrated *in vacuo* until a gummy residue was obtained. The residue was taken up in acetone/water (9:1, 500 mL) and poured into rapidly stirring water (2.50 L). The crude lignin was filtered off and dried under vacuum. Next, the dried lignin was taken up in acetone/methanol (9:1) and poured into rapidly stirring diethyl ether (2.00 L). The precipitated lignin was filtered off and dried under vacuum to give purified birch lignin (15.3 g).

The experimental results obtained with birch lignin as starting material are summarized in Table S4. For reaction conditions and procedures, see section 3.3.

Table S4. Combination of conditions using birch lignin as starting material.

entry	oxidation	cleavage	**17a** [%]	**17b** [%]	**17c** [%]	**17d** [%]	**17a-d** [%]
1	***t*BuONO**/DDQ	**Zn**/NH_4Cl	-	-	-	-	-
2	***t*BuONO**/DDQ	$\mathbf{H_2NNH_2}$/KOH	0.2	0.28	0.43	0.17	1.08
3	**NO**/DDQ	$\mathbf{H_2NNH_2}$/KOH	0.07	0.15	0.47	0.18	0.87
4	**NO**/DDQ	**Zn**/NH_4Cl	-	-	-	-	-

Yields determined after purification by column chromatography using maleic acid as internal standard. Yields based on birch lignin.

6. Characterization of oxidation and cleavage products

4-Hydroxy-3-methoxybenzaldehyde (vanillin) (23a)

MW: 152.15 g/mol

The oxidation reaction was performed according to the general procedure for oxidation reactions with NO/DDQ.

^{1}H NMR (400 MHz, DMSO-d_6): δ (ppm) = 9.77 (s, 1 H), 7.42 (dd, *J* = 8.1 Hz, *J* = 1.9 Hz 1 H), 7.39 (d, *J* = 1.9 Hz 1 H), 6.69 (d, *J* = 8.1 Hz, 1 H), 3.84 (s, 3 H).

^{1}H NMR (400 MHz, $CDCl_3$): δ (ppm) = 9.83 (s, 1 H), 7.42-7.45 (m, 2 H), 7.05 (d, *J* = 8.5 Hz, 1 H), 3.98 (s, 3 H).

The analytical data obtained is in agreement with those reported in literature.[5]

4-Hydroxy- 3,5-dimethoxybenzaldehyde (syringaldehyde) (23b)

MW: 182.18 g/mol

The oxidation reaction was performed according to the general procedure for oxidation reactions with NO/DDQ.

^{1}H NMR (400 MHz, DMSO-d_6): δ (ppm) = 9.77 (s, 1 H), 7.21 (s, 2 H), 3.84 (s, 6 H).

^{1}H NMR (400 MHz, $CDCl_3$): δ (ppm) 9.83 (s, 1 H), 7.16 (s, 2 H), 3.98 (s, 6 H).

The analytical data obtained is in agreement with those reported in literature.[6]

2-Methoxyphenol (17a)

MW: 124.14 g/mol

The cleavage reaction was performed according to the general procedure for cleavage reactions.

^{1}H NMR (400 MHz, DMSO-d_6): δ (ppm) = 8.89 (s, 1 H), 6.92 – 6.88 (m, 1 H), 6.78 – 6.70 (m, 3 H), 3.74 (s, 3 H).

The analytical data obtained is in agreement with those reported in literature.[7]

2-Methoxy-4-methylphenol (17b)

MW: 138.17 g/mol

The cleavage reaction was performed according to the general procedure for cleavage reactions.

^{1}H NMR (400 MHz, DMSO-d_6): δ (ppm) = 8.63 (s, 1 H), 6.72 (d, *J* = 2.0 Hz, 1 H), 6.63 (d, *J* = 7.9 Hz, 1 H), 6.53 (ddd, *J* = 7.9, *J* = 2.0, *J* = 0.8 Hz, 1 H), 3.72 (s, 3 H), 2.19 (s, 3 H).

The analytical data obtained is in agreement with those of a commercially available sample.

^{1}H NMR (400 MHz, $CDCl_3$): δ (ppm) = 6.8 (d, *J* = 7.9 Hz, 1 H), 6.6 – 6.7 (m, 2 H), 3.9 (s, 6 H), 2.3 (d, *J* = 0.7 Hz, 3 H).

The analytical data obtained is in agreement with those reported in literature.[8]

2,6-Dimethoxyphenol (17c)

MW: 154.17 g/mol

The cleavage reaction was performed according to the general procedure for cleavage reactions.

^{1}H NMR (400 MHz, DMSO-d_6): δ (ppm) = 8.26 (s, 1 H), 6.69 (dd, *J* = 9.1, *J* = 7.3 Hz, 1 H), 6.59 (d, *J* = 7.8 Hz, 2 H), 3.74 (s, 6 H).

The analytical data obtained is in agreement with those reported in literature.[9]

2,6-Dimethoxy-4-methylphenol (17d)

MW: 168.19 g/mol

The cleavage reaction was performed according to the general procedure for cleavage reactions.

^{1}H NMR (400 MHz, DMSO-d_6): δ (ppm) = 8.00 (s, 1 H), 6.41 (s, 2 H), 3.72 (s, 6 H), 2.20 (s, 3 H).

The analytical data obtained is in agreement with those of a commercially available sample.

^{1}H NMR (400 MHz, $CDCl_3$): δ (ppm) = 6.4 (d, *J* = 0.7 Hz, 2 H), 3.8 (s, 6 H), 2.3 (t, *J* = 0.6, 0.6 Hz, 3 H).

The analytical data obtained is in agreement with those reported in literature.[10]

7. NMR-spectra

Oxidation products

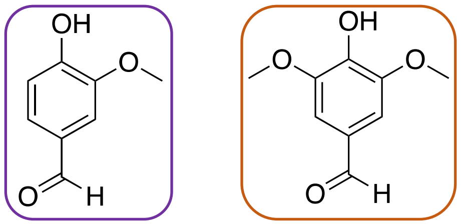

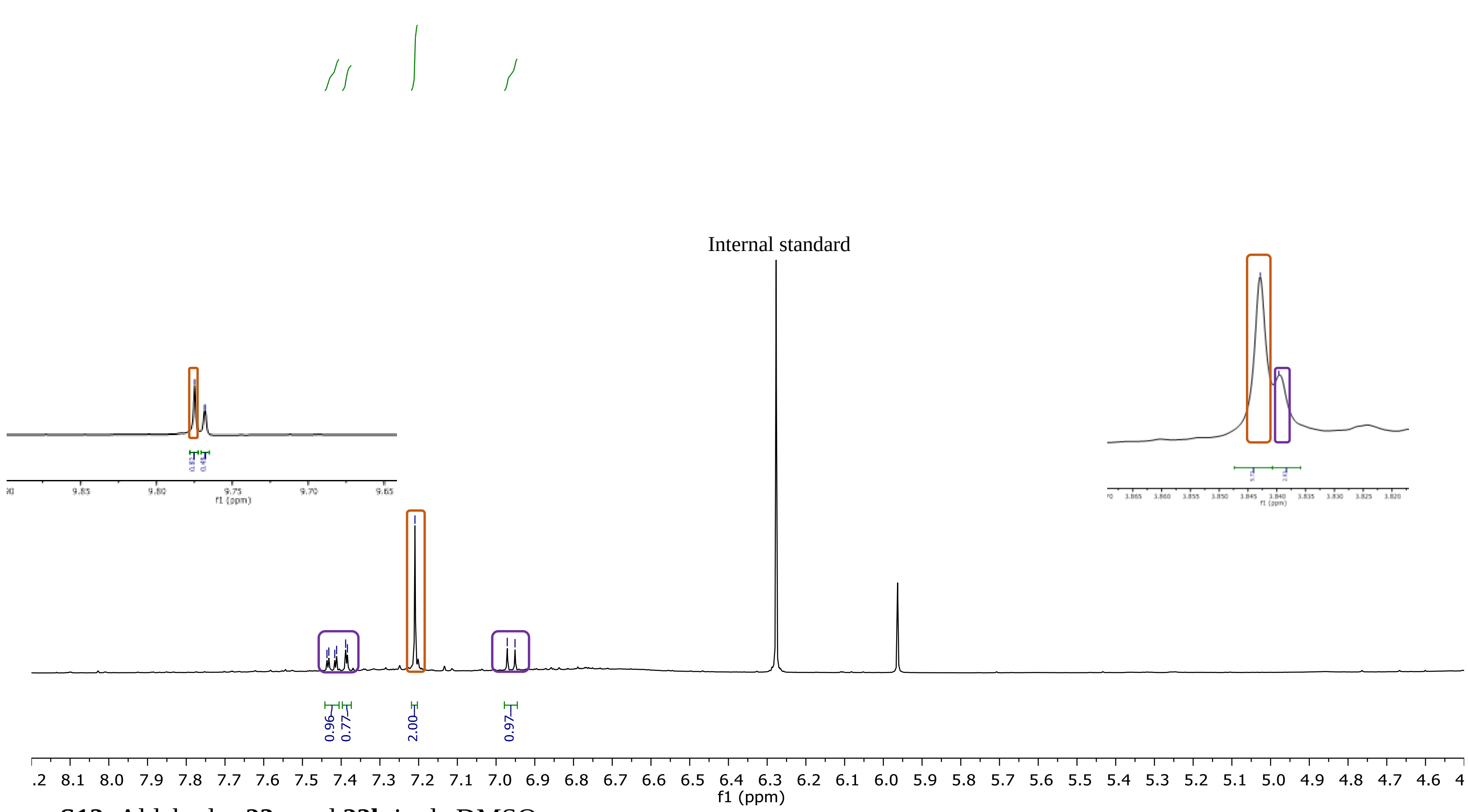

Figure S12. Aldehydes **23a** and **23b** in d_6-DMSO

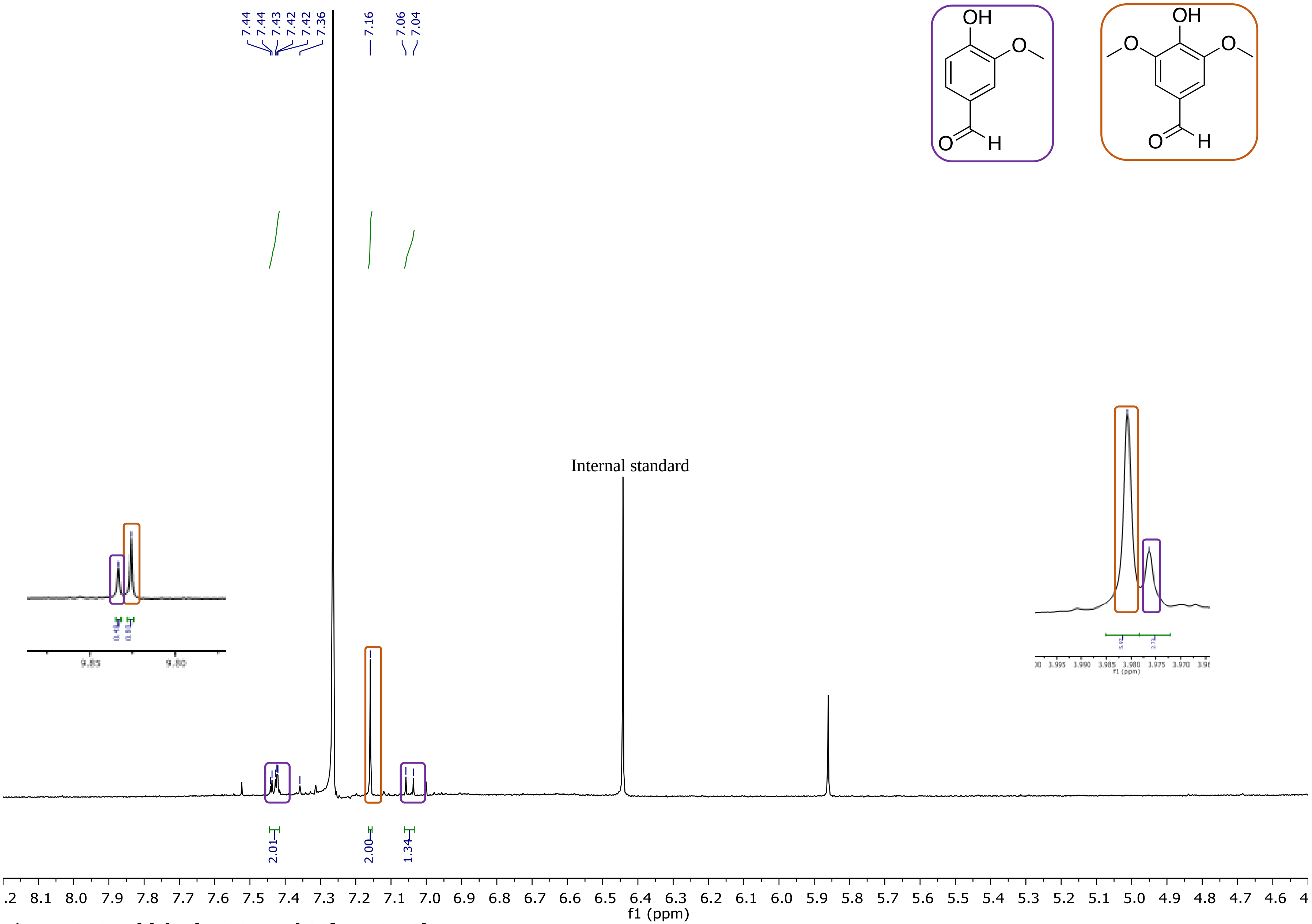

Figure S13. Aldehydes **23a** and **23b** in $CDCl_3$

Spectra of the oxidation products used as reference compounds

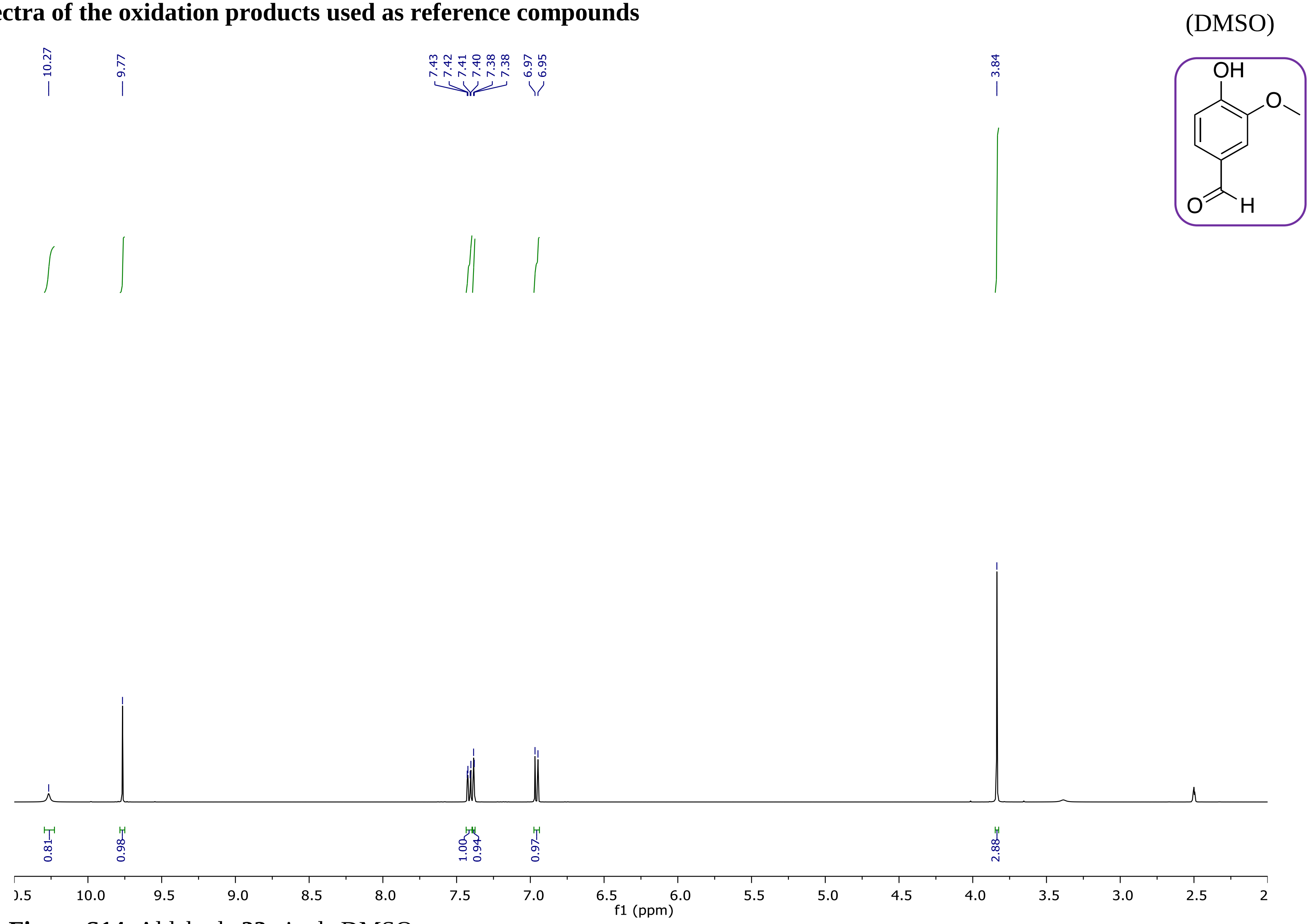

Figure S14. Aldehyde **23a** in d_6-DMSO.

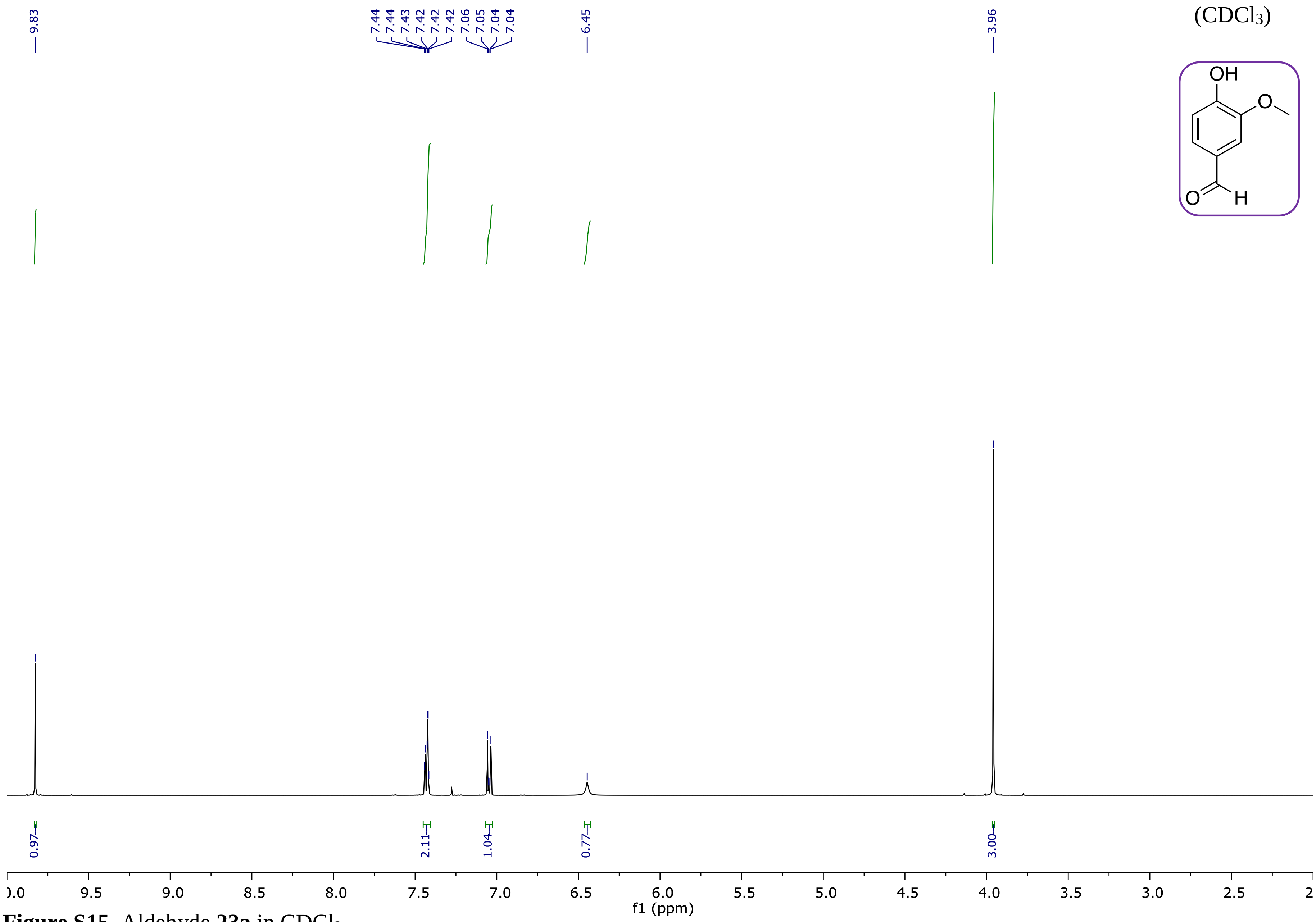

Figure S15. Aldehyde **23a** in $CDCl_3$.

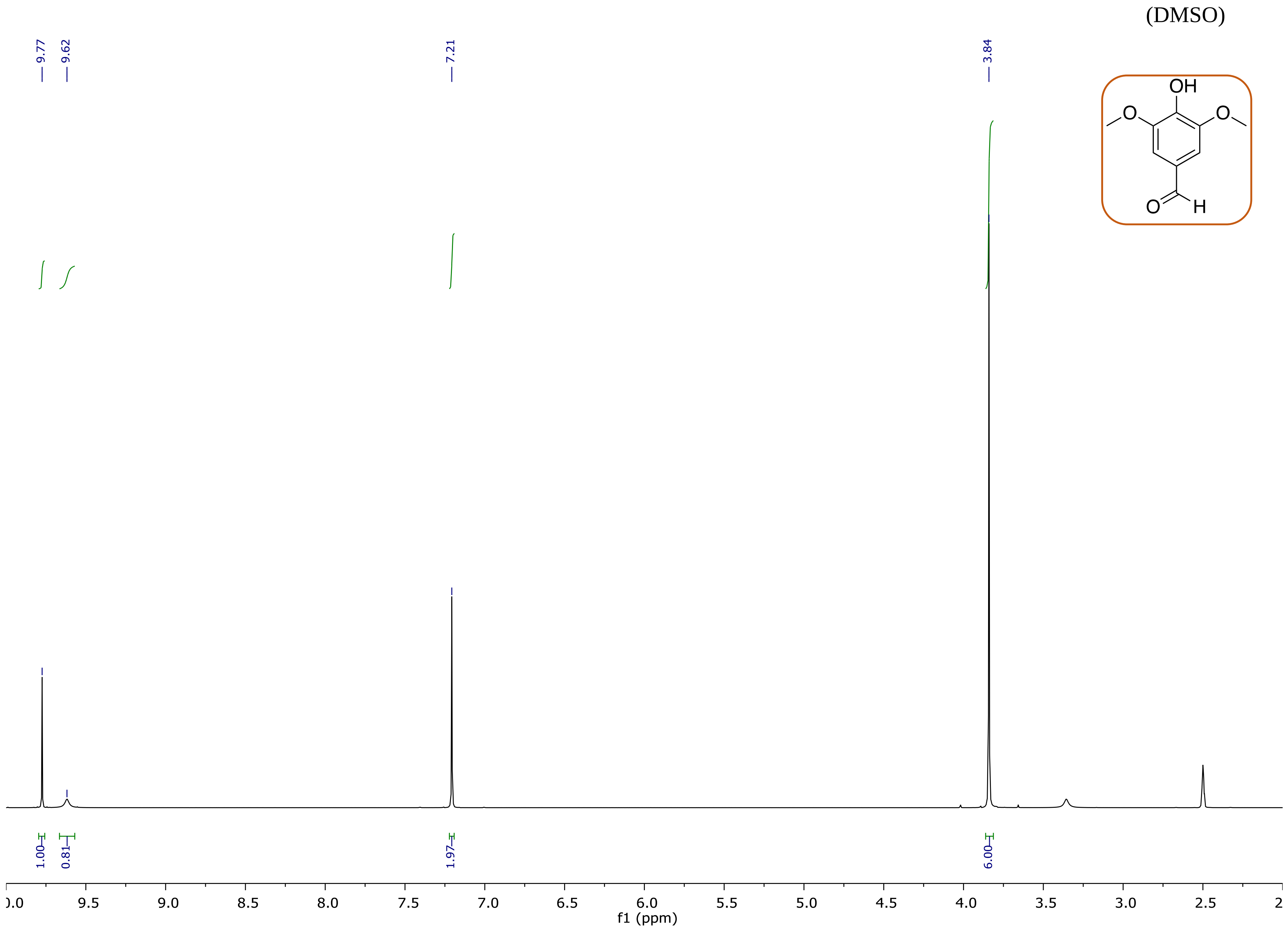

Figure S16. Aldehyde **23b** in d_6-DMSO.

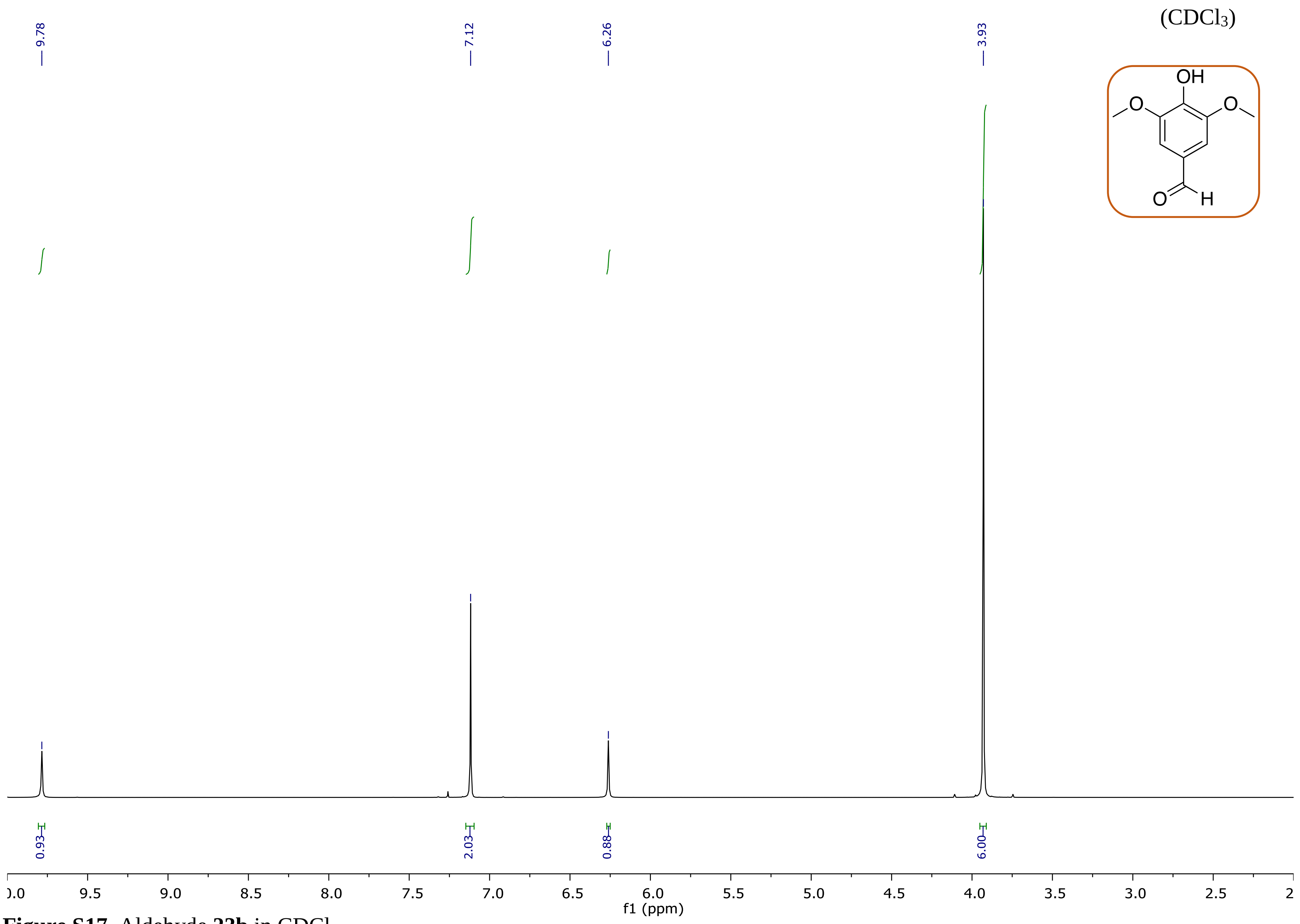

Figure S17. Aldehyde **23b** in $CDCl_3$.

Reductive cleavage products

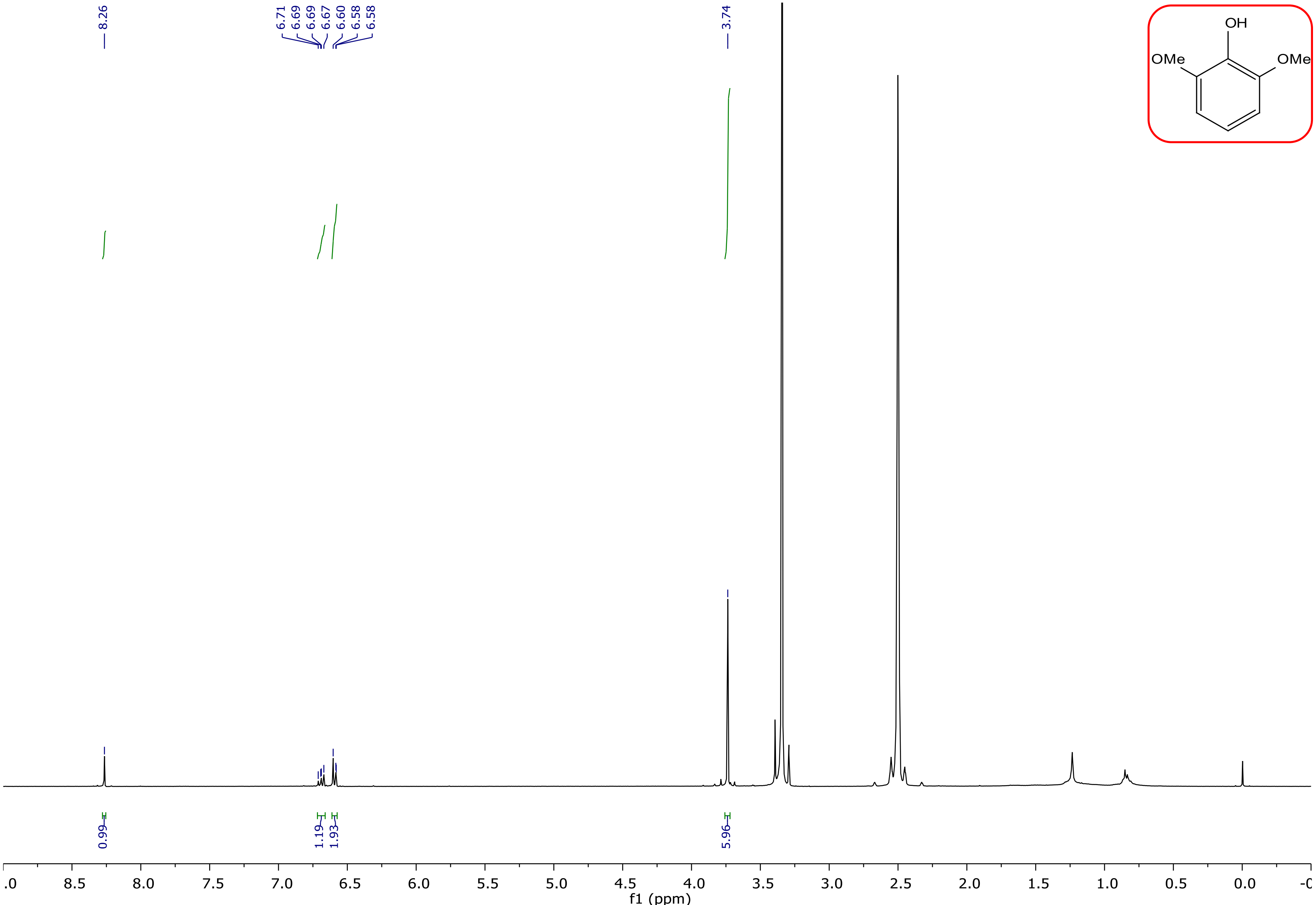

Figure S18. Phenol **17c** in d_6-DMSO.

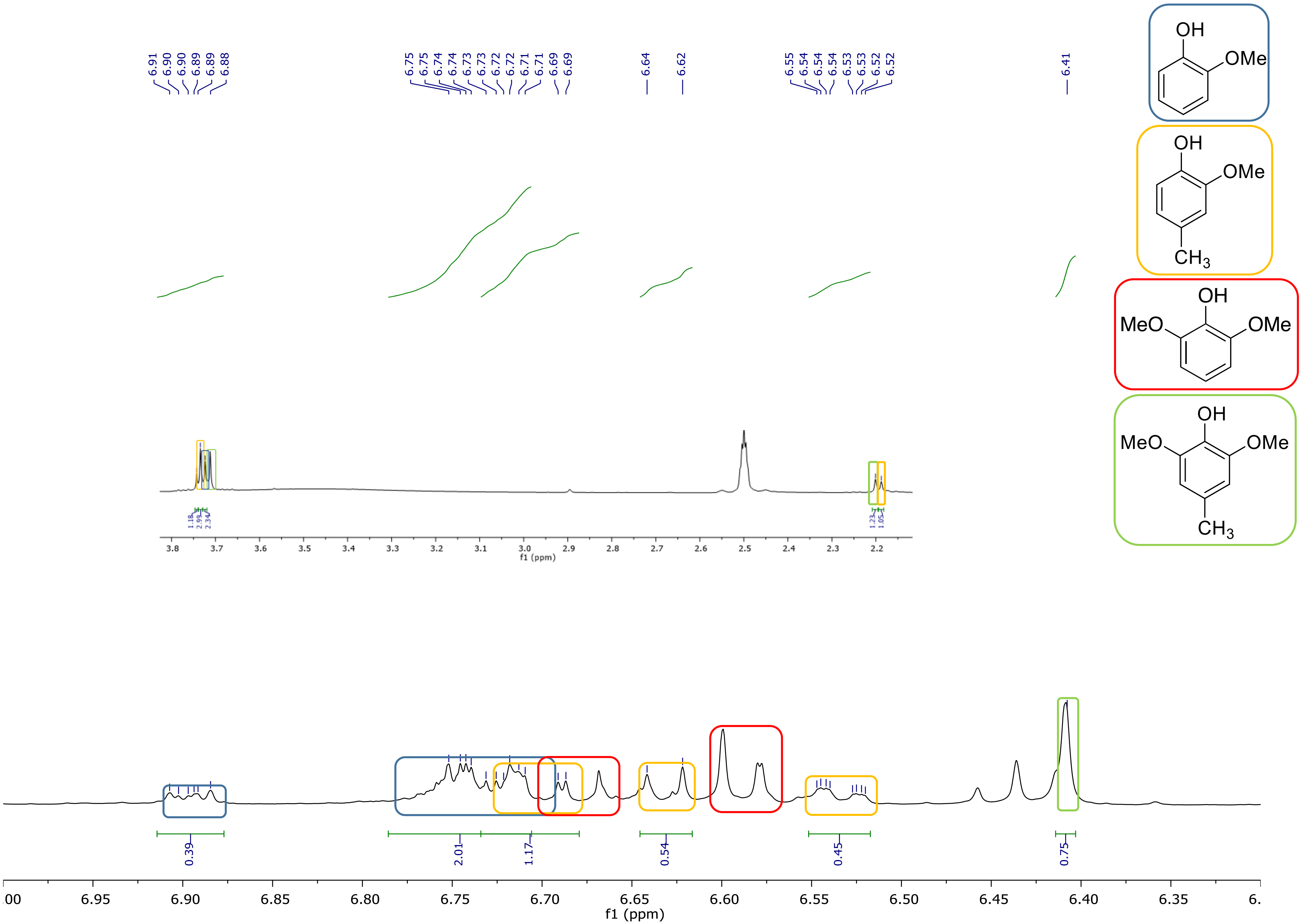

Figure S19. Phenols **17a-d** in d_6-DMSO.

Examples for spectra obtained from the cleavage experiments summarized in Figure 4

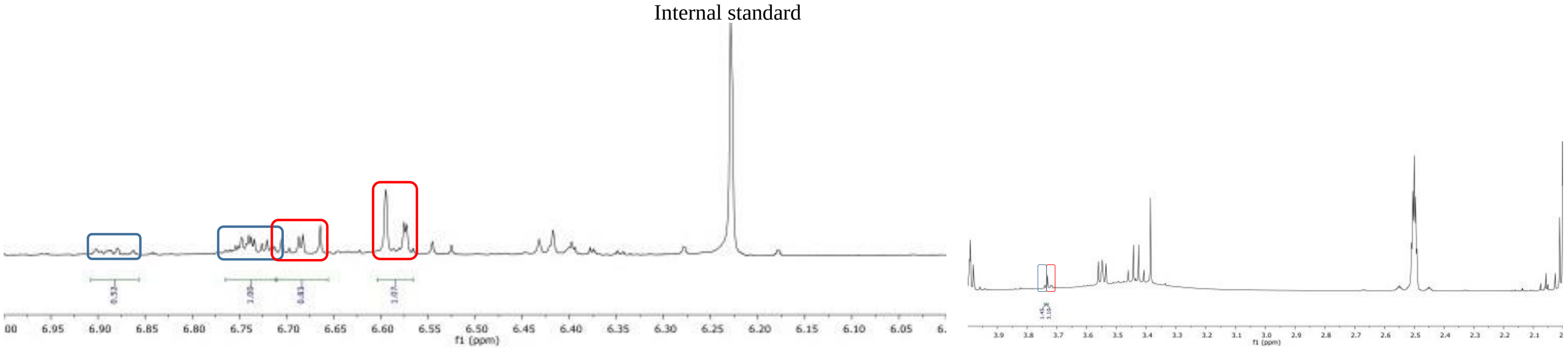

Figure S20. NMR spectrum for Figure 4, entry 2

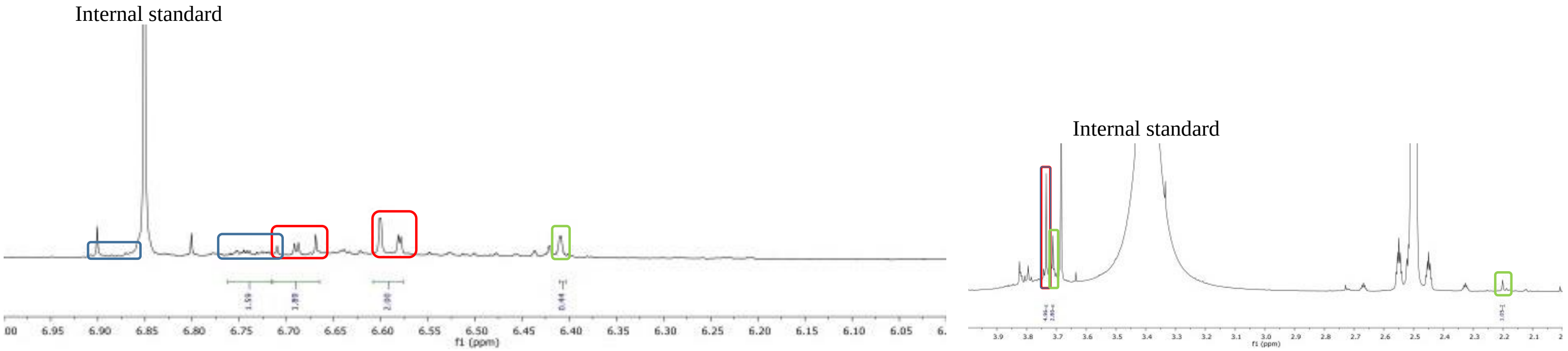

Figure S21. NMR spectrum for Figure 4, entry 6

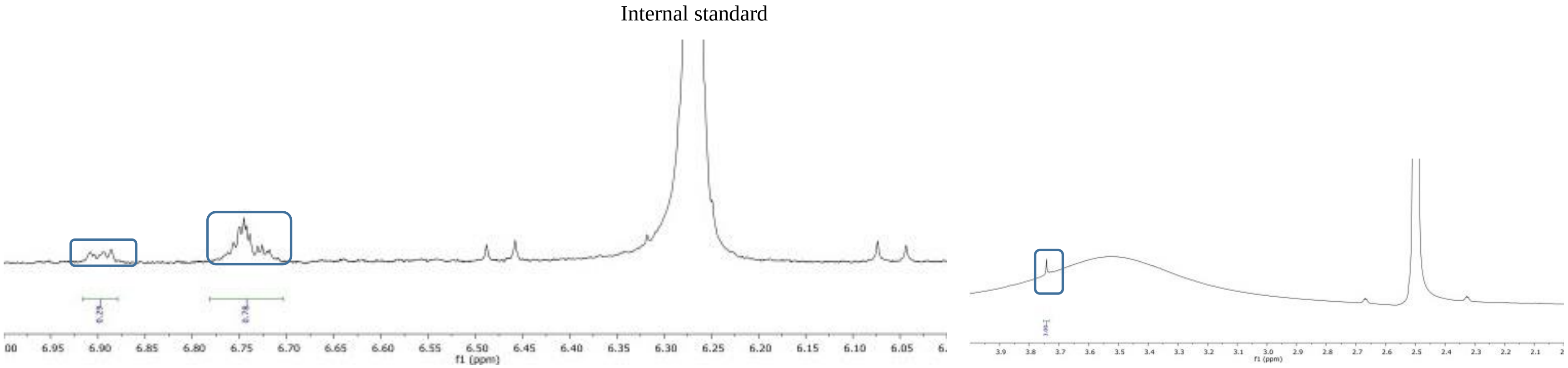

Figure S22. NMR spectrum for Figure 4 entry 9

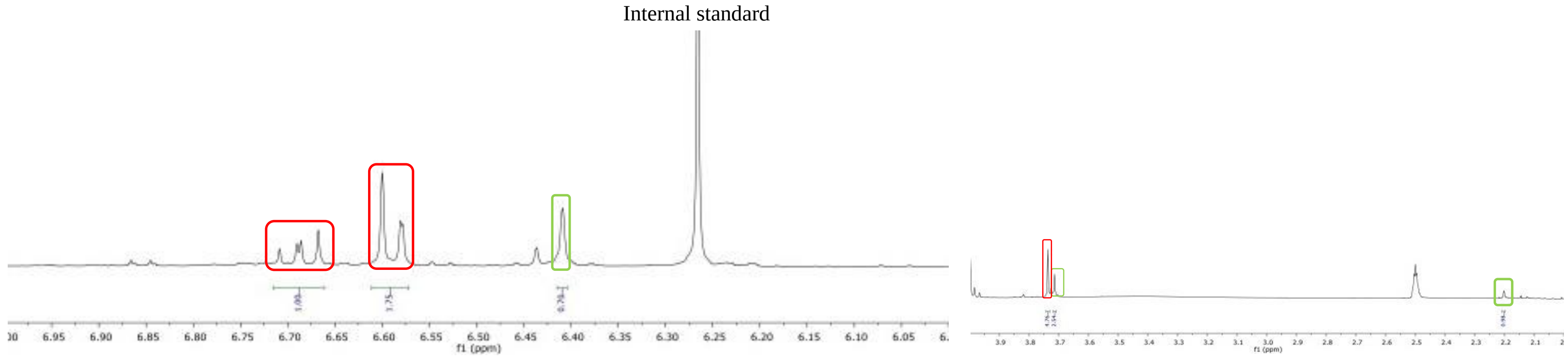

Figure S23. NMR spectrum for Figure 4 entry 11

Spectra of cleavage products used as reference compounds

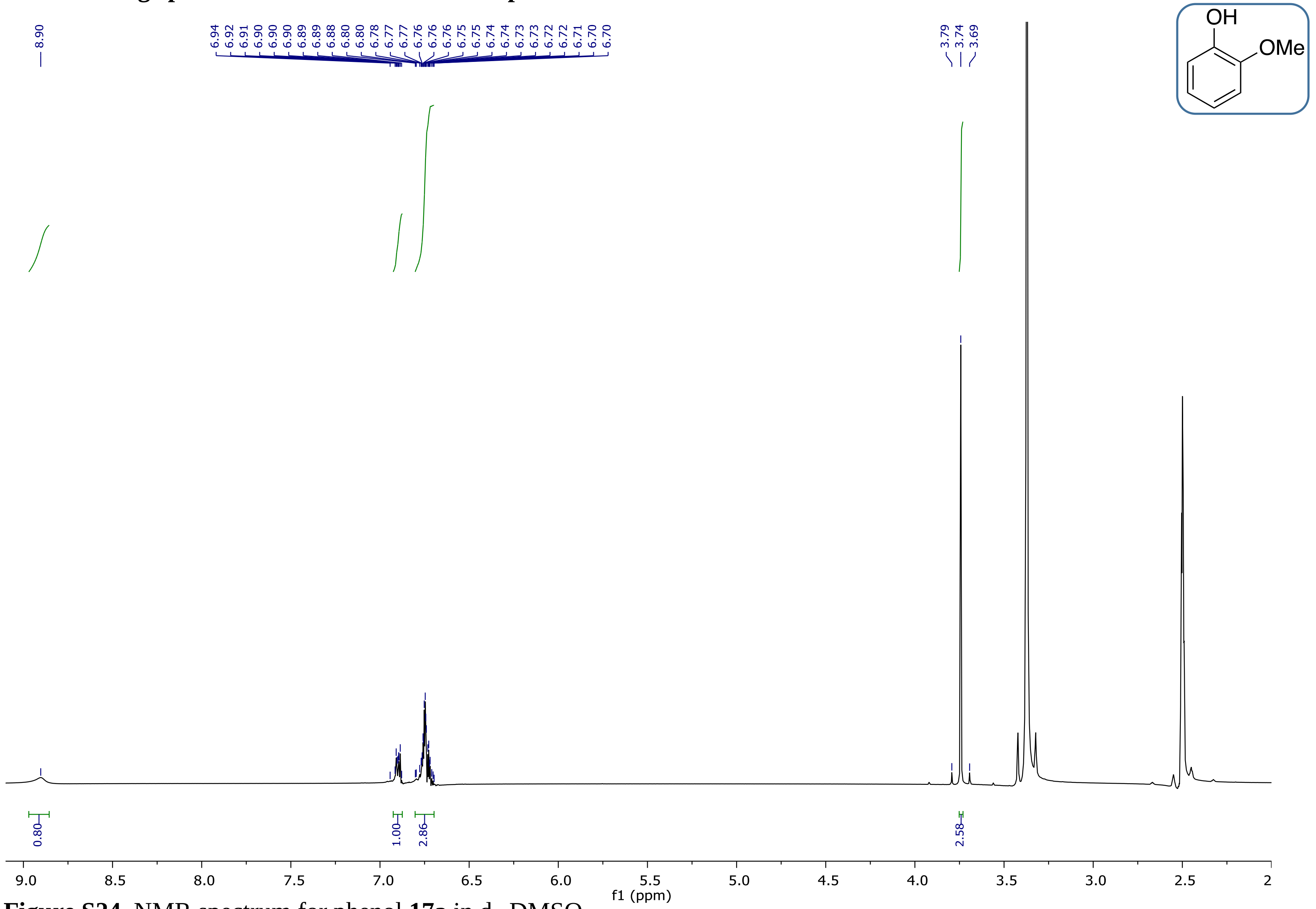

Figure S24. NMR spectrum for phenol **17a** in d_6-DMSO

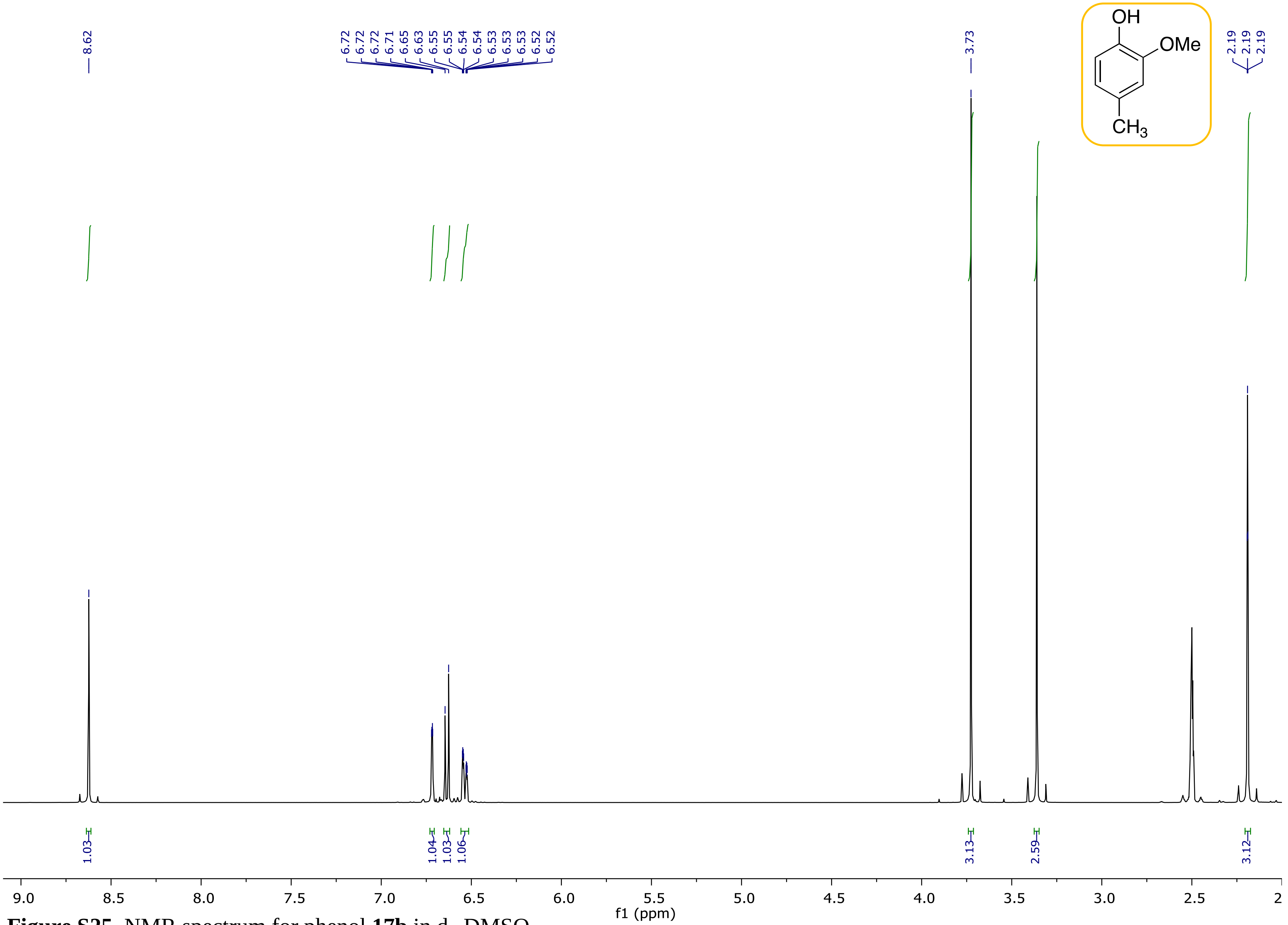

Figure S25. NMR spectrum for phenol **17b** in d_6-DMSO

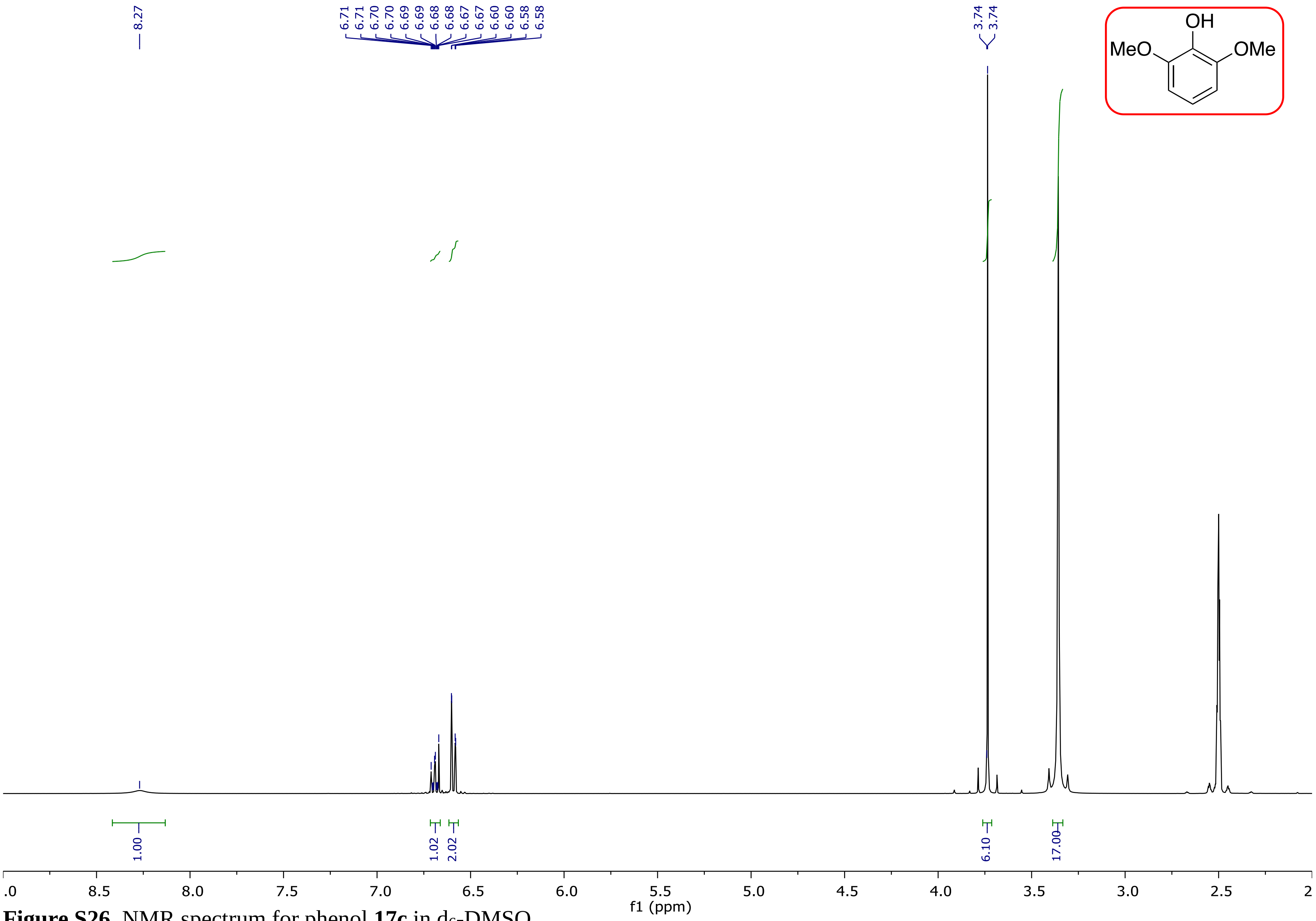

Figure S26. NMR spectrum for phenol **17c** in d_6-DMSO

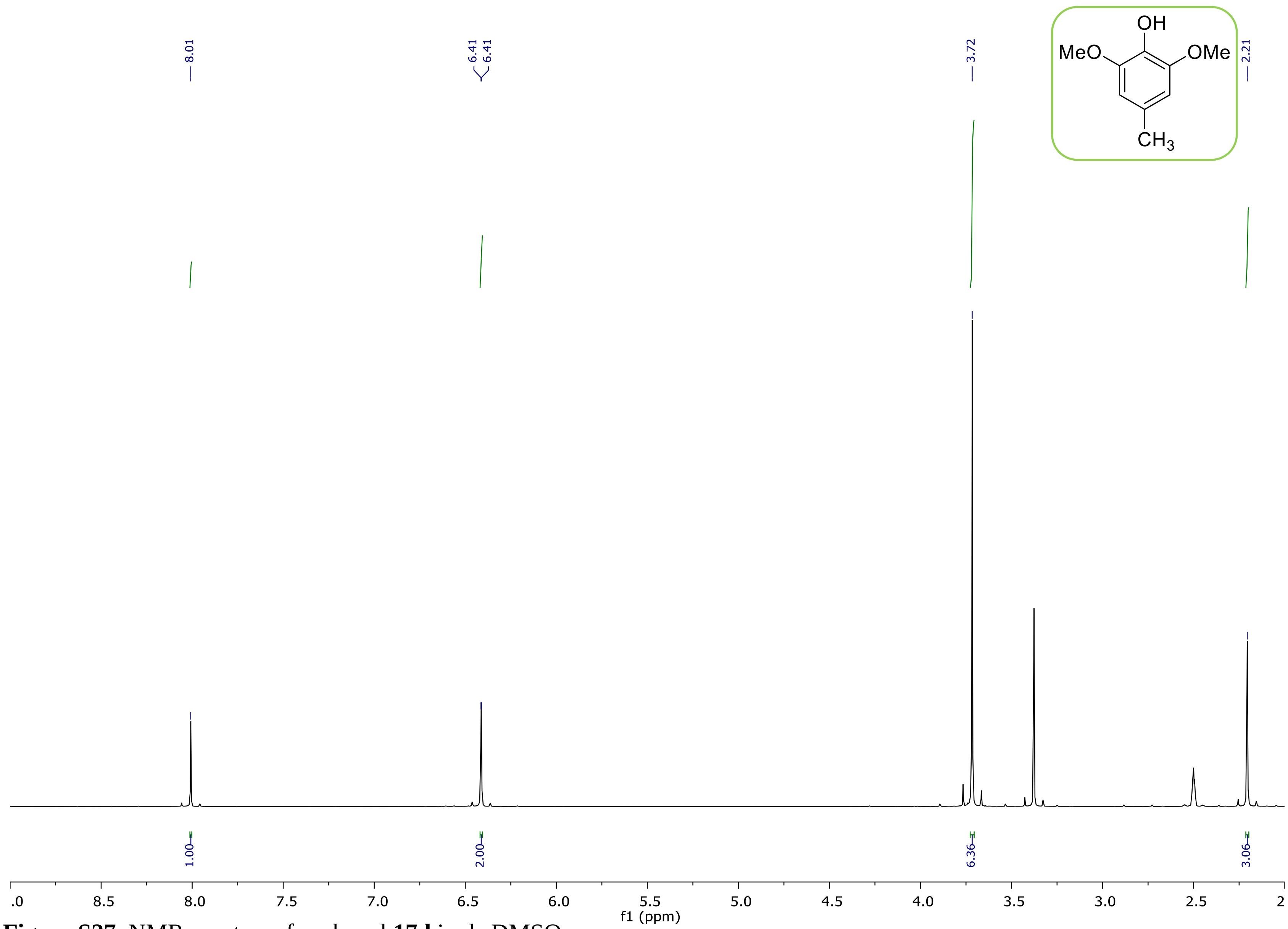

Figure S27. NMR spectrum for phenol **17d** in d_6-DMSO

Spectra of control reactions

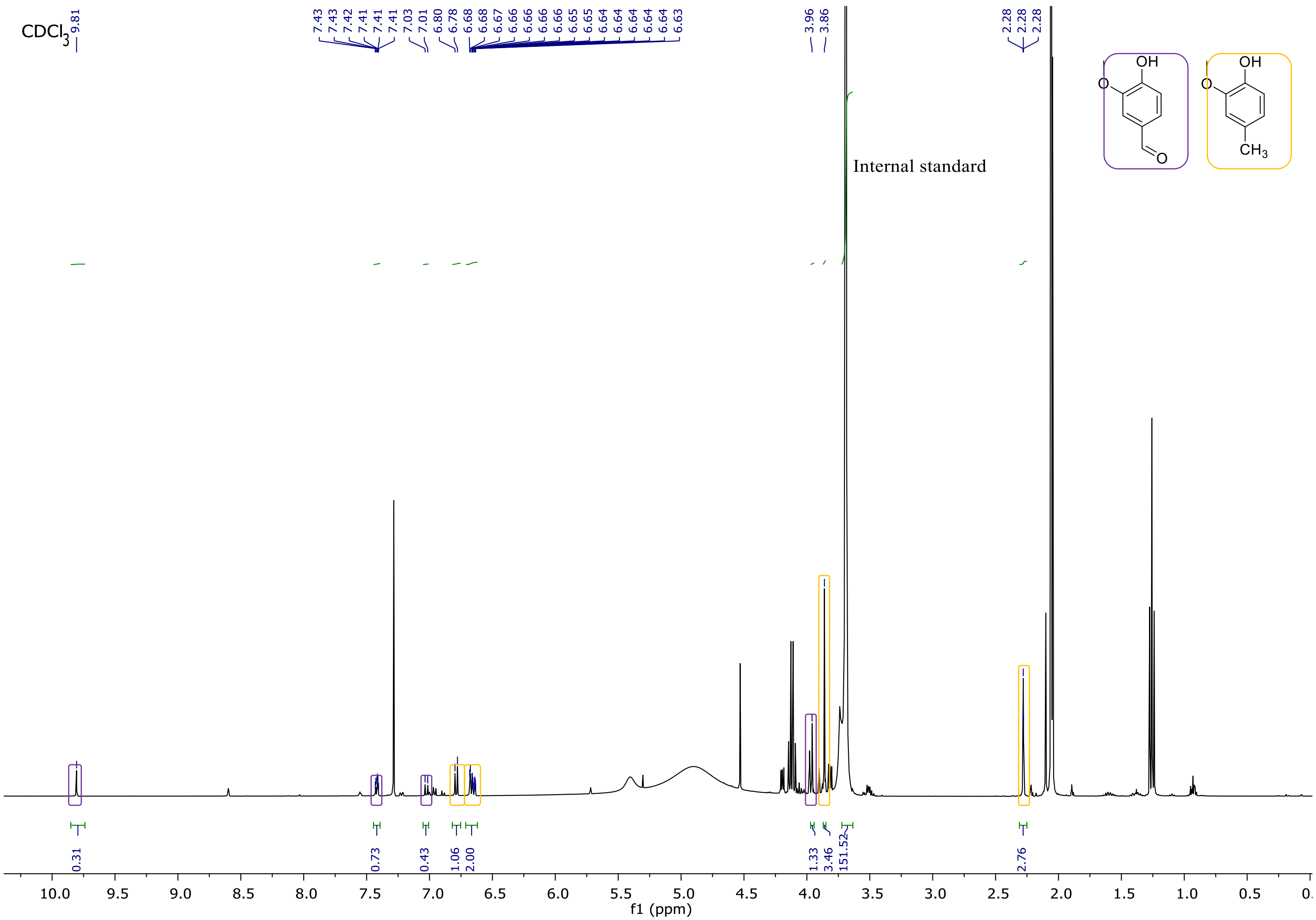

Figure S28. Reduction of vanillin (**23a**)

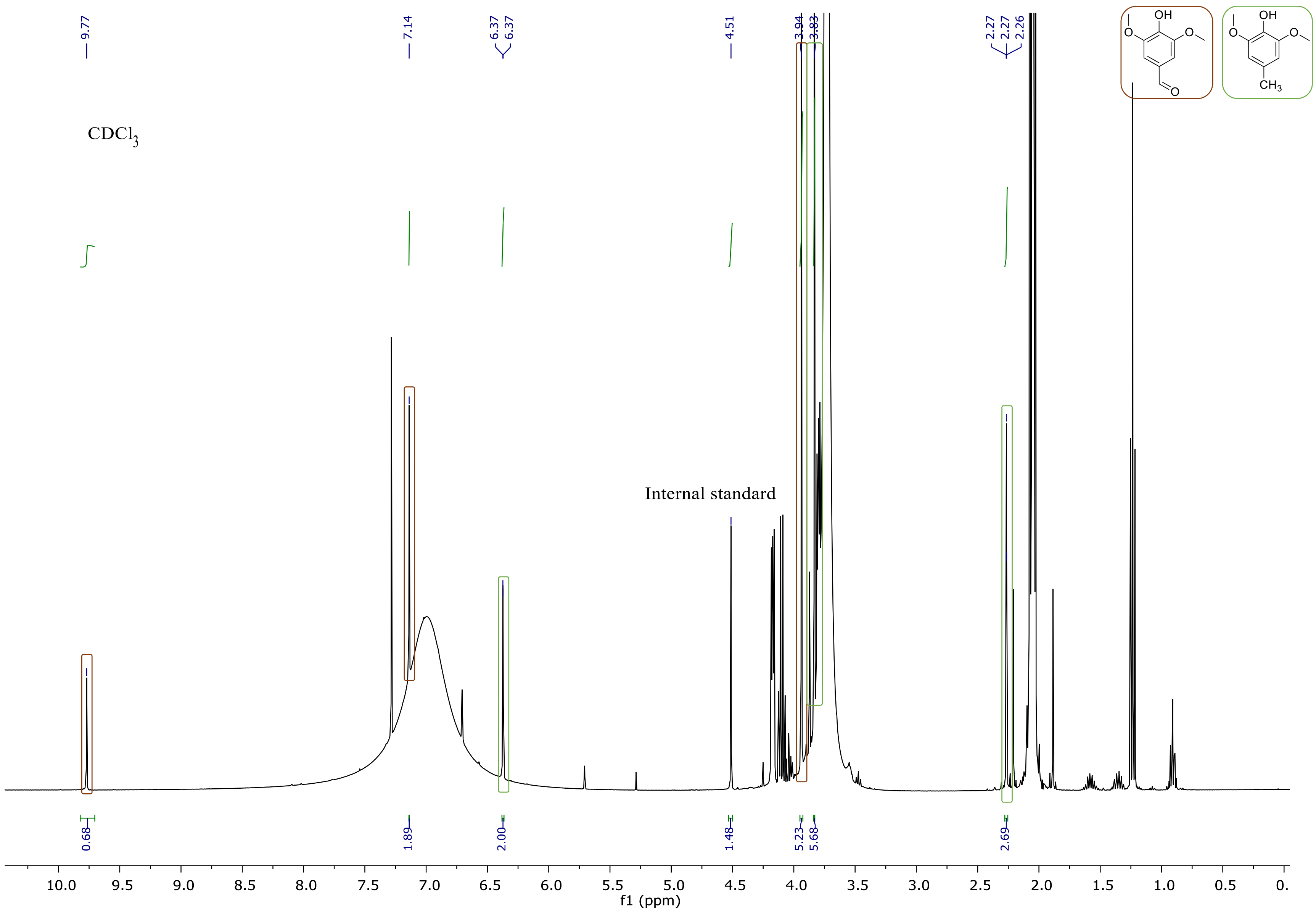

Figure S29. Reduction of syringaldehyde (**23b**)

8. References

(1) Fraunhofer-Zentrum für Chemisch-Biotechnologische Prozesse CBP, Leuna for providing organosolv lignin.

(2) *Lehrbuch der Anorganischen Chemie*. 33rd ed.; Holleman, A. F., Wiberg, E., Ed.; Walter de Gruyter & Co.: Berlin, 1985; pp 91–100.

(3) Sheldon, R. A. The E factor: fifteen years on. *Green Chem*. **2007**, *9*, 1273–1283.

(4) Lancefield, C. S.; Ojo, O. S.; Tran, F.; Westwood, N. J. Isolation of functionalized phenolic monomers through selective oxidation and C-O bond cleavage of the β-O-4 linkages in lignin. *Angew. Chem., Int. Ed*. **2015**, *54*, 258–262.

(5) (a) For $CDCl_3$ data see: Bao, K.; Fan, A.; Dai, Y.; Zhang, L.; Zhang, W.; Cheng, M.; Yao, X. Selective demethylation and debenzylation of aryl ethers by magnesium iodide under solvent-free conditions and its application to the total synthesis of natural products. *Org. Biomol. Chem.* **2009**, *7*, 5084–5090. (b) For DMSO data see: Azarpira, A.; Lu, F.; Ralph, J. Reactions of dehydrodiferulates with ammonia. *Org. Biomol. Chem.* **2011**, *9*, 6779–6787.

(6) (a) For $CDCl_3$ data see: Jiang, J.-A.; Chen, C.; Huang, J.-G.; Liu, H.-W.; Cao, S.; Ji, Y.-F. $Cu(OAc)_2$-catalyzed remote benzylic $C(sp^3)$–H oxyfunctionalization for C=O formation directed by the hindered para-hydroxyl group with ambient air as the terminal oxidant under ligand- and additive-free conditions. *Green Chem.* **2014**, *16*, 1248–1254. (b) For DMSO data see: Kärkäs, M. D.; Bosque, I.; Matsuura, B. S.; Stephenson, C. R. J. Photocatalytic Oxidation of Lignin Model Systems by Merging Visible-Light Photoredox and Palladium Catalysis, *Org. Lett.* **2016**, *18*, 5166–5169.

(7) Yu, C.-W.; Chen, G. S.; Huang, C.-W.; Chern, J.-W. Efficient Microwave-Assisted Pd-Catalyzed Hydroxylation of Aryl Chlorides in the Presence of Carbonate. *Org. Lett.* **2012**, *14*, 3688–3691.

(8) Bakos, M.; Gyömöre, A.; Domján, A.; Soós, T. Auto-Tandem Catalysis with Frustrated Lewis Pairs for Reductive Etherification of Aldehydes and Ketones. *Angew. Chem. Int. Ed.* **2017**, *56*, 5217–5221.

(9) Cheung, C. W.; Buchwald, S. L. Palladium-Catalyzed Hydroxylation of Aryl and Heteroaryl Halides Enabled by the Use of a Palladacycle Precatalyst. *J. Org. Chem.* **2014**, *79*, 5351–5358.

(10) Guo, Y.; Ji, S.-Z.; Chen, C.; Liu, H.-W.; Zhao, J.-H.; Zheng, Y.-L.; Ji, Y.-F. A ligand-free, powerful, and practical method for methoxylation of unactivated aryl bromides by use of the CuCl/HCOOMe/MeONa/MeOH system. *Res. Chem. Intermed.* **2015**, *41*, 8651–8664.
